# Oral microalgae-ROS-responsive hierarchical targeting delivery system for precision therapy of ulcerative colitis

**DOI:** 10.1186/s12951-026-04890-5

**Published:** 2026-08-06

**Authors:** Ru Feng, Yongle Zhao, Xiang Sun, Luke Ge, Pengqi Zhu, Wanying Zhang, Caifang Gao, Jinghua Sun, Ruiping Zhang

**Affiliations:** 1 Shanxi Bethune Hospital, Tongji Shanxi Hospital, Shanxi Academy of Medical Sciences, Third Hospital of Shanxi Medical University, Taiyuan, 030032 China; 2 Department of Radiology, Shanxi Provincial People’s Hospital, Fifth Hospital of Shanxi Medical University, Taiyuan, 030000 China; 3 First Hospital of Shanxi Medical University, Taiyuan, 030001 China

**Keywords:** Purple radish anthocyanin, Spirulina platensis, ROS-responsive, Mitochondria-targeted, Ulcerative colitis

## Abstract

Ulcerative colitis (UC) is a chronic, non-specific inflammatory bowel disease. Currently, the lack of precise oral colon-targeted delivery systems prevents current drugs from effectively accumulating at inflamed sites, leading to unsatisfactory therapeutic outcomes. Although anthocyanins possess great potential to mitigate oxidative damage and inflammation cascade, their poor gastrointestinal stability and oral absorption limit clinical application. To address this challenge, we innovatively propose a hierarchical targeted nano-delivery system (SP@PRA@PCT) that combines Spirulina platensis (SP) with reactive oxygen species (ROS)-responsive nanoparticles loaded with purple radish anthocyanin (PRA), aiming to enhance gastrointestinal stability and achieve precise retention in inflamed regions. SP@PRA@PCT exhibits hierarchical targeting properties: the unique helical morphology of SP facilitates its retention in intestinal villi, thereby prolonging intestinal residence time; the SP-loaded PRA@PCT (with an encapsulation efficiency of 75.5% and a drug loading content of 4.03%) can further target intracellular mitochondria and specifically release PRA in response to ROS within the inflammatory microenvironment. In vitro studies demonstrate that this system effectively scavenges excessive ROS, alleviates mitochondrial dysfunction, promotes epithelial cell migration, regulates the expression of inflammatory cytokines, and inhibits apoptosis. On DSS-induced colitis in mice, SP@PRA@PCT significantly prolongs intestinal retention time to 48 h and achieves superior therapeutic efficacy compared to free PRA or non-targeted controls, as evidenced by preserved colon length, decreased disease activity index, restored intestinal barrier integrity, and effective modulation of gut microbiota balance through increased abundance of beneficial bacteria. In summary, this oral microalgae-based ROS-responsive nano-delivery system, which integrates ROS-responsive release, mitochondrial targeting, and microalgae-mediated bioadhesion, effectively overcomes the key bottleneck of poor oral absorption, providing a highly promising strategy for the oral delivery of natural extracts to manage colitis.

**Graphical Abstract:**

**Figure:**
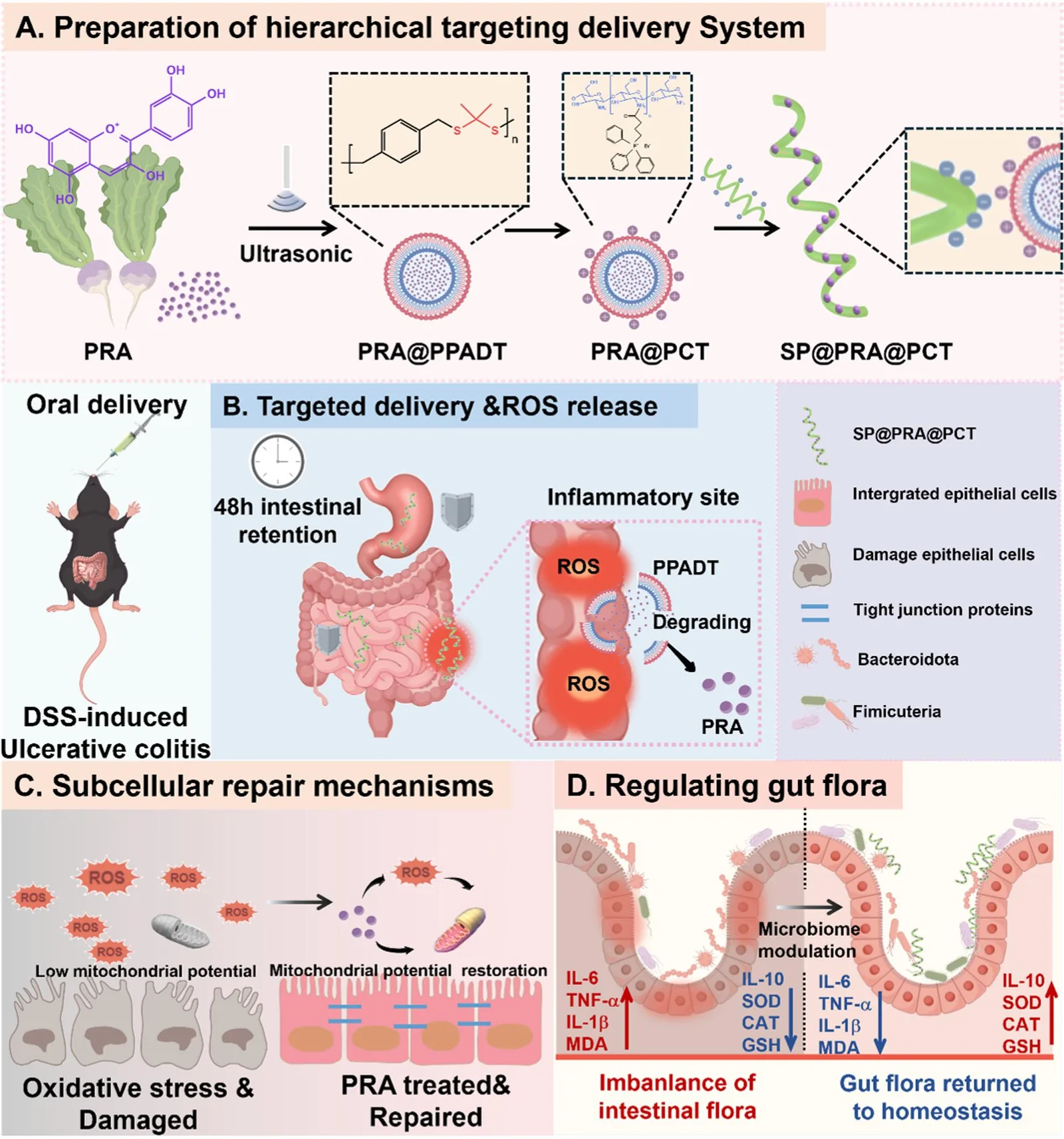

**Supplementary Information:**

The online version contains supplementary material available at https://doi.org/10.1186/s12951-026-04890-5.

## Introduction

Ulcerative colitis (UC) is a chronic, non-specific inflammatory bowel disease. Its characteristics of being difficult to cure and prone to recurrence significantly reduces patient quality of life and elevates the risk of colorectal cancer [1–3]. With the rising global incidence of UC, this disease has become a major public health concern [4, 5]. The pathogenesis of UC is complex, involving multiple processes such as excessive activation of oxidative stress, impaired intestinal barrier function, immune system dysregulation, and intestinal flora disturbance [6–8]. Current clinical management of UC primarily relies on drugs, including 5-aminosalicylic acid (5-ASA), immunosuppressants, and biologics. However, these therapeutic approaches still face challenges such as serious systemic side effects, limited efficacy, and insufficient drug accumulation at inflamed sites [9–11]. In recent years, natural plant extracts have provided new perspectives for UC treatment due to their high biocompatibility and multi-target action properties. Components such as curcumin, resveratrol, and anthocyanins have been confirmed to possess various activities, including anti-inflammatory, antioxidant, and immunomodulatory effects [12, 13]. Among them, anthocyanins have shown great potential in protecting the intestinal epithelial barrier, modulating immune function, scavenging free radicals, and regulating gut microbiota [14, 15]. Anthocyanins from black corn, black goji berries, and purple sweet potatoes can alleviate mouse colitis by inhibiting pyroptosis and modulating the gut microbiota structure [16–18]. Compared with other anthocyanins, purple radish anthocyanins (PRA) feature abundant raw material supply, significantly lower extraction costs, and a well-established extraction process, rendering them a viable candidate for development as dietary supplements or long-term adjunctive therapeutic agents. However, the poor gastrointestinal stability, oral absorption, and lack of targeting of anthocyanins themselves severely restrict their clinical translation and application [19, 20].

The emergence of nano-drug delivery systems offers a promising strategy to enhance the stability, oral absorption and targeting ability of anthocyanins. Li et al. [21] prepared a succinylated casein-phospholipid-anthocyanin nanosystem (CLS) via one-step self-assembly, effectively overcoming the low bioavailability of anthocyanins and significantly alleviating acute/chronic lung injury. Yuan et al. [22] employed a layer-by-layer self-assembly method to encapsulate proanthocyanidins into a casein core, innovatively developing a CCH@PC multifunctional nanoparticle system that breaks through the traditional limitations of poor stability and low absorption of proanthocyanidins. In contrast to the above conventional drug delivery systems, intelligent responsive delivery system can achieve precise drug release triggered by specific signals (such as pH, enzymes, and reactive oxygen species (ROS)) within the lesion microenvironment. During the pathological progression of UC, the excessive accumulation of ROS at inflamed sites serves as a key mediator of tissue damage, meanwhile, correlates closely with disease severity, making ROS an ideal “triggering switch” for drug release. Mitochondria, as the primary energy hub of intestinal epithelial cells, are the main source of intracellular ROS, and their dysfunction impairs oxidative phosphorylation (OXPHOS), reduces adenosine triphosphate (ATP) production, and drives the accumulation of excessive ROS, thereby disrupting barrier integrity and exacerbating the progression of intestinal inflammation in UC [23, 24]. Given that direct delivery of antioxidants to mitochondria can effectively scavenge local ROS and protect mitochondrial function, thereby interrupting the oxidative stress cascade at its source, integrating a mitochondria-targeting strategy into ROS-responsive nanocarriers represents a rational design approach to maximize therapeutic efficacy in UC treatment. Poly (1,4-phenyleneacetone dimethylene thioketal) (PPADT) is a polymer containing ROS-sensitive thioketal bonds, exhibiting excellent stability under acidic, alkaline, and protease-rich conditions [25]. Therefore, we employ PPADT to encapsulate PRA, which enhances the stability of PRA and prevents their degradation in the gastrointestinal environment, meanwhile, enables mitochondria-targeted delivery by coating the nanoparticle surface with TPP-modified chitosan (CS-TPP). Ultimately, this system achieves site-specific release of anthocyanins under high ROS level conditions.

Although the aforementioned ROS-responsive nanoparticles effectively address the issues of gastrointestinal stability and site-specific drug release at inflamed sites, achieving long-term inflammatory colon-targeted retention is particularly crucial for oral treatment of UC [26, 27]. Spirulina platensis (SP), a natural microalga, has garnered significant attention due to its unique helical morphology, which enables physical interlocking with intestinal villi, thereby significantly prolonging intestinal retention time and enhancing local drug concentration [28, 29]. Lei et al. [30] developed a novel oral drug delivery system (SP@CSC) that combines natural microcarrier SP with chitosan-functionalized ceria (CSC) nanogels. The acid resistance and intestinal retention properties of SP improved the distribution of CSC nanoparticles in the intestine and prolonged their residence time, thereby promoting targeted antioxidant effects. This provides a promising strategy for improving drug absorption and treating intestinal diseases. Meanwhile, Wang et al. [31] designed an EcN@PDA@HP biohybrid robot that integrates pressure-responsive microalgae, probiotics, and an intestinal braking system, achieving efficient oral delivery and precise targeting of active ingredients, offering a revolutionary approach for the treatment of UC. Furthermore, as a dietary supplement, SP is rich in bioactive components such as proteins and polysaccharides, and possesses superior antioxidant, anti-inflammatory, and gut microbiota-modulating functions [32, 33]. Therefore, it is regarded as an ideal candidate that combines carrier functionality with adjunctive therapeutic effects.

**Scheme 1:**
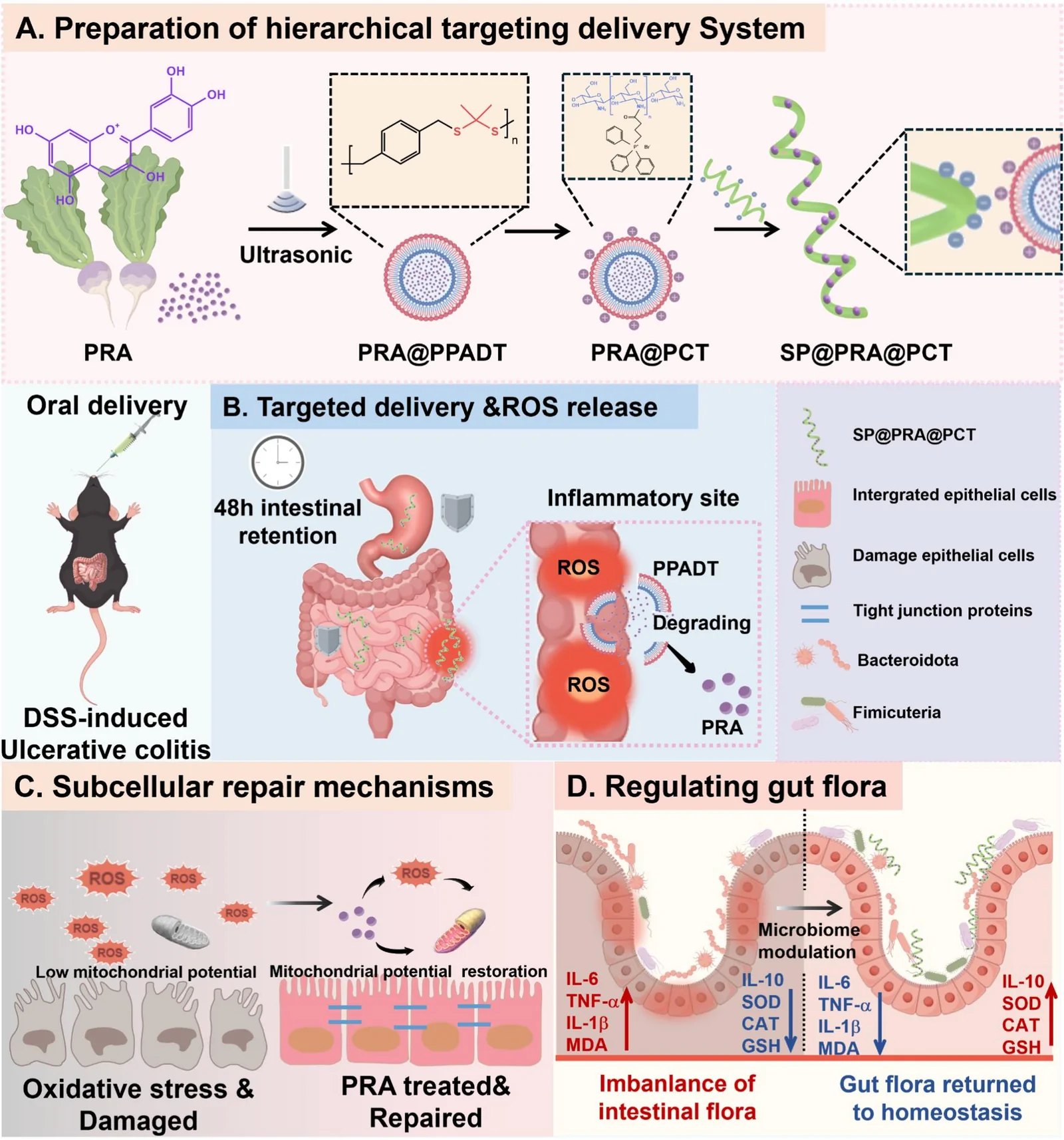
Schematic illustration of a SP-based ROS-responsive oral nano-delivery system (SP@PRA@PCT) for the treatment of ulcerative colitis. (**A**) The preparation process of SP@PRA@PCT. (**B-D**) Anti-colitis efficacy and mechanisms of SP@PRA@PCT system: leveraging SP-mediated intestinal retention and ROS-responsive release, precisely delivers anthocyanins to colonic inflammatory sites. Through mitochondrial targeting, it scavenges ROS, restores cellular function, and modulates inflammation and gut microbiota, thereby significantly alleviating colitis.

In this study, the ROS-responsive polymer PPADT was used to encapsulate PRA, and the surface was modified with CS-TPP to obtain mitochondria-targeting nanoparticles (PRA@PCT). Subsequently, these nanoparticles were electrostatically anchored onto the surface of the helical microalga SP to construct the SP@PRA@PCT delivery system, aiming to achieve hierarchical targeting and synergistic therapy for UC (Scheme 1). Our results demonstrate that SP@PRA@PCT exhibits hierarchical targeting properties: the unique helical morphology of SP enables specific capture and adhesion to intestinal villi, significantly prolonging intestinal retention time; SP retained in the inflamed colonic region modulate the gut microbiota; meanwhile, the SP-loaded PRA@PCT further targets intracellular mitochondria and specifically responds to ROS in the inflammatory microenvironment to trigger the release of PRA. The released PRA effectively scavenges excessive ROS, alleviates mitochondrial dysfunction, regulates the expression of inflammatory cytokines, and inhibits apoptosis, thereby significantly ameliorating intestinal injury. In summary, SP@PRA@PCT integrates ROS-responsive, mitochondria-targeted properties with microalgae-mediated bioadhesion, successfully overcoming the key limitation of low oral absorption of PRA. With excellent biosafety, this system provides a promising and translatable oral strategy for the treatment of UC through anti-inflammatory, antioxidant, and microbiota-regulating mechanisms.

## Results and discussion

### Synthesis and characterization of SP@PRA@PCT

PRA were extracted from purple radish using a combined solvent extraction and ultrasound-assisted protocol. HPLC analysis identified cyanidin as the predominant monomer, accounting for over 89% of the total anthocyanin content (Fig. S1 and Table S2). Morphological characterization revealed that the extracted PRA existed as particulate matter with an average size of approximately 5 nm (Fig. S2).

**Fig. 1:**
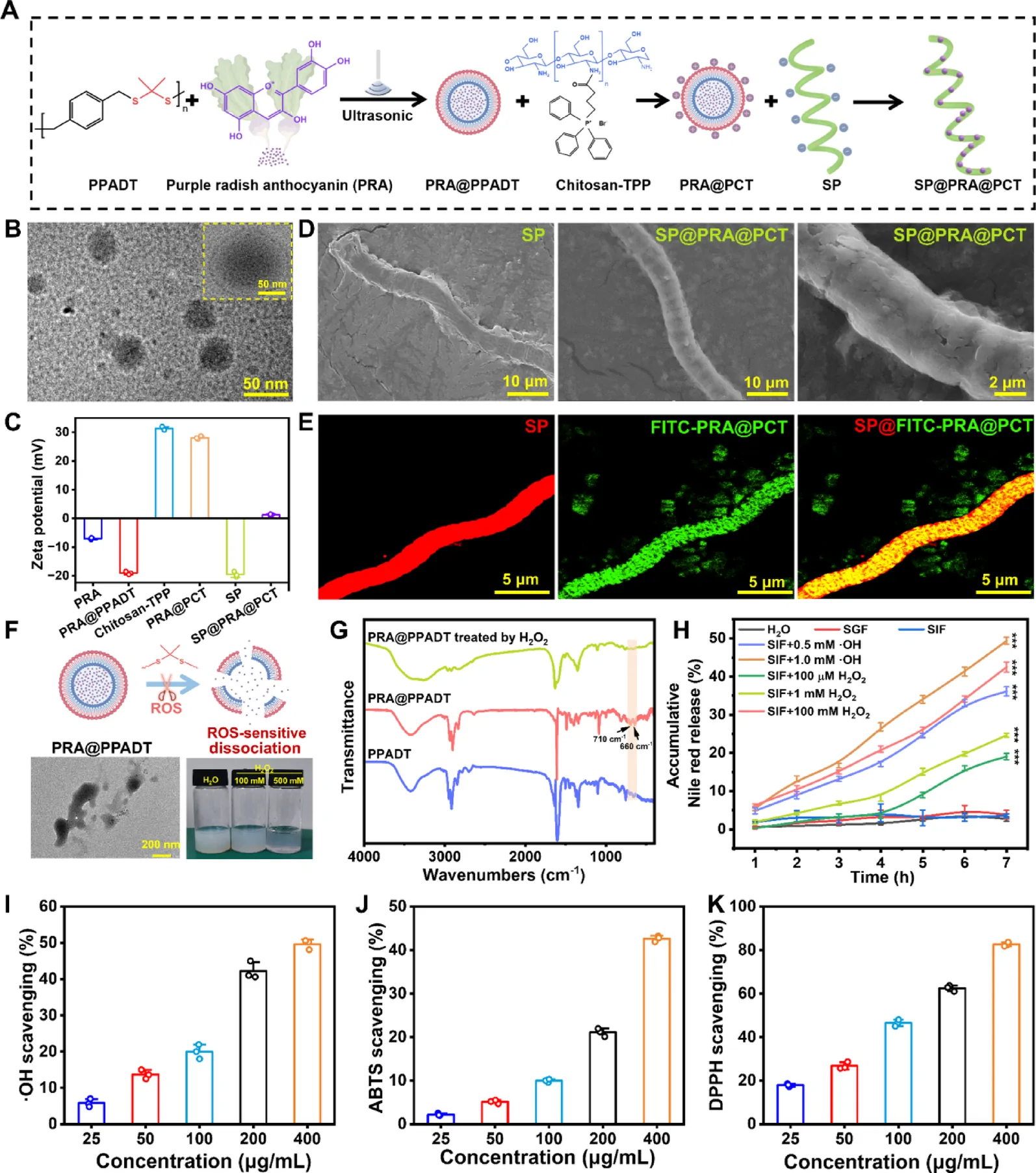
Preparation and characterization of SP@PRA@PCT. (**A**) Schematic illustration of the synthetic route for SP@PRA@PCT. (**B**) TEM image of PRA@PPADT. Scale bar = 50 nm. (**C**) Zeta potentials of the as-prepared samples. (**D**) SEM image of SP@PRA@PCT. (**E**) CLSM image of SP@PRA@PCT. (**F**) Schematic of the ROS-responsive disassembly of PRA@PPADT; TEM image of PRA@PPADT after incubation with H_2_O_2_ showing the release of PRA; photograph demonstrating the change in solution transmittance upon reaction. (**G**) FTIR spectra of PPADT, PRA@PPADT, and PRA@PPADT after H_2_O_2_ treatment. (H) Cumulative release profiles of Nile Red-loaded PRA@PCT nanoparticles following incubation with ·OH and H_2_O_2_ at varying concentrations. (**I-K**) ·OH, ABTS^+^, and DPPH scavenging assay of PRA@PCT at different concentration. Data are mean ± SD (*n* = 3). *p < 0.05, **p < 0.01, ***p < 0.001

The ROS-responsive polymer PPADT was synthesized according to a previously reported procedure [25]. As shown in Fig. S3, the characteristic proton resonances at 6.16 ppm, 4.00 ppm, and 4.07 ppm in the ^1^H NMR spectrum matched well with literature values, verifying the successful synthesis of PPADT [34]. Subsequently, PRA-loaded PPADT nanoparticles (PRA@PPADT) were fabricated via a water-in-oil-in-water (w/o/w) emulsion-solvent evaporation method. By investigating the effect of four oil phase-to-external water phase volume ratios (1:1, 1:2, 1:4, and 1:10) on the encapsulation efficiency of PRA, the preparation conditions of PRA@PPADT were systematically optimized. The results showed that the corresponding encapsulation efficiency values were 32.6%, 75.5%, 69.2%, and 36.1%, respectively, with the highest encapsulation efficiency (75.5%) achieved at a volume ratio of 1:2, at which the nanoparticles exhibited a uniform spherical morphology (Figs. S4 and S5). Based on these results, the volume ratio of 1:2 was selected as the optimal preparation ratio, and the drug loading of PRA@PPADT was determined to be 4.03%. TEM image (Fig. 1B) and hydrodynamic size distribution analysis revealed that the as-prepared PRA@PPADT nanoparticles possessed a uniform spherical morphology with an average diameter of approximately 91 nm (Fig. S6). The zeta potential of PRA@PPADT was determined to be −18.3 ± 0.4 mV.

To enable efficient mitochondrion-targeting ability and electrostatic assembly with negatively charged SP (zeta potential: −19.80 ± 1.43 mV), the nanoparticles were surface-functionalized with CS-TPP. Successful conjugation of CS and TPP via amide bond formation was confirmed by ^1^H NMR (Fig. S7), UV-Vis spectroscopy (Figure S8A), and FTIR spectroscopy (Fig. S8B) [35]. The resulting CS-TPP conjugate exhibited a positive zeta potential of 30.8 ± 0.3 mV. Following surface modification, the PRA@PCT nanoparticles displayed a positively charged surface with a zeta potential of 27.60 ± 0.2 mV (Fig. 1C), which not only enhanced the colloidal stability of the nanoparticles but also facilitated their electrostatic adsorption onto the SP surface.

Morphological examination revealed that SP exhibited a typical helical structure with lengths ranging from 100 to 500 μm (Fig. S9). Such a microstructure is beneficial for anchoring onto intestinal villi, thereby enhancing physical retention within the gastrointestinal tract [31, 36]. SEM images clearly demonstrated that the surface of SP was uniformly and densely coated with PRA@PCT nanoparticles (Fig. 1D). Furthermore, CLSM imaging using FITC-labeled PRA@PCT showed abundant green-fluorescent PRA@PCT nanoparticles firmly attached to the red-fluorescent SP surface (Fig. 1E). TEM images further confirmed the extensive coverage of PRA@PCT nanoparticles on the SP surface (Fig. S10). Collectively, these results validate the successful fabrication of the SP@PRA@PCT delivery system.

To investigate the ROS-responsive release behavior, PRA@PCT nanoparticles were treated with H_2_O_2_ solution. TEM image revealed that PRA@PPADT underwent dissociation and subsequent PRA release upon exposure to H_2_O_2_ (Fig. 1F). FTIR spectroscopy demonstrated that both PPADT and PRA@PPADT exhibited characteristic thioketal absorption peaks at 660 cm^−1^ and 710 cm^−1^, which disappeared after H_2_O_2_ treatment, confirming that cleavage of the thioketal linkages led to the ROS-responsive dissociation of PRA@PCT (Fig. 1G) [37].

To further evaluate the stability and ROS-responsive release behavior of the nanoparticle nano-delivery system, Nile Red was employed as a hydrophobic fluorescent probe to monitor fluorescence intensity changes under various ROS concentrations. Given that Nile Red fluorescence is strongly quenched in aqueous media, its fluorescence intensity variation serves as a reliable indicator of nanoparticle disassembly and cargo release [23, 38]. In pure water and simulated gastrointestinal fluids, the fluorescence intensity remained almost unchanged over 7 h. These results collectively confirmed the robust gastrointestinal stability of PRA@PCT, supporting their suitability for oral delivery (Figs. 1H and S11A). In contrast, within the H_2_O_2_ concentration range of 100 µM to 100 mM, the fluorescence intensity exhibited a negative correlation with both H_2_O_2_ concentration and treatment time. Moreover, the rate of fluorescence decrease accelerated with increasing H_2_O_2_ concentrations, and the release percentage of Nile Red in 100 mM H_2_O_2_ exceeded 42.5%, confirming the concentration-dependent dissociation of PRA@PCT (Fig. 1H). Similarly, upon treatment with 0.5 mM to 1 mM ·OH, a sustained increase in Nile Red release was observed, further validating the ROS-responsive release behavior of the nano-delivery system. Furthermore, nanoparticle dissociation reduced light scattering, leading to increased transmittance of the suspension (Figure S11A). Digital photographs showed a marked increase in the transmittance of the PRA@PPADT suspension after exposure to H_2_O_2_ and ·OH, providing direct visual evidence of the ROS-responsive dissociation of the nanoparticles. More importantly, direct quantification of PRA release via UV-Vis spectrophotometry in simulated intestinal fluid with 100 µM H_2_O_2_ further corroborated the ROS-triggered drug release profile (Figure S11C), which was highly consistent with the release trend of Nile Red.

The in vitro antioxidant activity of PRA@PCT was systematically evaluated using ·OH, ABTS^+^, and DPPH radical scavenging assays. As illustrated in Figs. 1I-K, PRA@PCT exerted concentration-dependent radical scavenging effects across the tested concentration range (25–400 µg/mL), with scavenging capacities increasing progressively with concentration. At 400 µg/mL, the scavenging efficiencies against ABTS^+^ and ·OH reached 42.6 ± 0.67% and 49.6 ± 1.34%, respectively, while the DPPH scavenging efficiency was as high as 82.6 ± 0.9%. Moreover, UV-Vis spectral analysis of the DPPH reaction system (Fig. S12) showed a progressive attenuation of the characteristic absorption peak at approximately 520 nm with increasing PRA@PCT concentration, further confirming efficient radical scavenging. These results collectively demonstrate that the PRA@PCT nanoparticle exhibits excellent antioxidant activity.

### Cellular validation of ROS-responsive release behavior

**Fig. 2:**
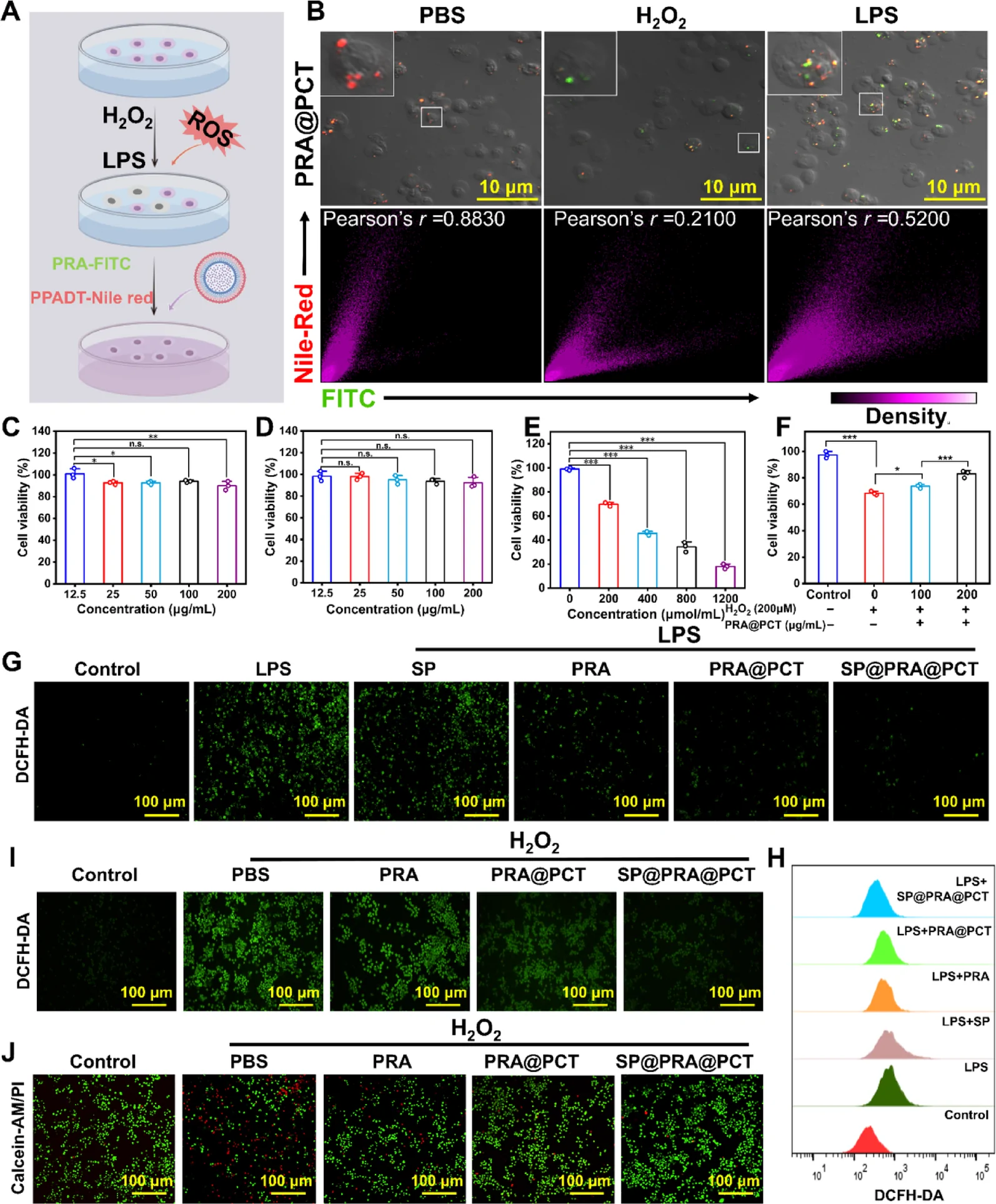
Cytotoxicity, ROS-responsive behavior, and antioxidant of SP@PRA@PCT. (**A**) Schematic representation of the ROS-responsive disassembly and cargo release process of FITC/Nile Red-dual-labeled PRA@PCT. (**B**) CLSM images of RAW 264.7 cells under PBS, H_2_O_2_, or LPS treatment (Red: Nile Red-labeled PPADT; Green: FITC-labeled PRA). (**C**) Viability of RAW 264.7 cells incubated with varying concentrations of PRA@PCT. (**D**) Viability of NCM 460 cells incubated with varying concentrations of PRA@PCT. (**E**) Influence of H_2_O_2_ concentration on the viability of RAW 264.7 cells. (**F**) Protective effects of PRA@PCT at different concentrations against H_2_O_2_-induced oxidative stress in RAW 264.7 cells. (**G**) Fluorescence microscopic images of intracellular ROS generation in LPS-stimulated RAW 264.7 cells. (**H**) Flow cytometric quantification of intracellular ROS levels in the indicated groups. (**I**) Fluorescence microscopic images of intracellular ROS levels in H_2_O_2_-stimulated RAW 264.7 cells. (**J**) Confocal live/dead staining images of H_2_O_2_-challenged RAW 264.7 cells. Data are mean ± SD (*n* = 3). *p < 0.05, **p < 0.01, ***p < 0.001, and n.s. = no significant

To investigate the ROS-responsive release behavior of PRA@PCT at the cellular level, we constructed FITC/Nile Red-PRA@PCT (Fig. 2A). In the FITC/Nile Red-PRA@PCT, PRA was labeled with FITC, while Nile Red was encapsulated within PPADT. When PPADT remained intact, Nile Red emitted red fluorescence. Upon ROS-induced degradation of PPADT, Nile Red fluorescence was quenched due to exposure to the aqueous environment, while FITC-labeled PRA was released. Consequently, the red and green fluorescence signals became spatially separated, visually reflecting the ROS-responsive dissociation of nanoparticles. To validate this responsive behavior, we treated RAW 264.7 cells with exogenous ROS (H_2_O_2_) and endogenous ROS induction conditions (LPS). After pretreatment with FITC|Nile Red-PRA@PCT for 3 h, RAW 264.7 cells were co-incubated with PBS (negative control), H_2_O_2_, or LPS for an additional 4 h. As shown in Fig. 2B, compared with the PBS-treated group, the red and green fluorescence signals were markedly separated in both H_2_O_2_ and LPS treatment groups. This change was further quantified by calculating the Pearson correlation coefficient between the red and green fluorescence channels using ImageJ: the coefficient was 0.88 in the PBS group, significantly decreased to 0.21 in the H_2_O_2_ group, and was 0.52 in the LPS group. These results confirm that ROS can effectively trigger the decomposition of PRA@PCT and the responsive release of PRA.

### The Biocompatibility and cellular protection of SP@PRA@PCT

To ensure the biocompatibility of the nanoparticles, the cytotoxicity of PRA@PCT against RAW 264.7 macrophages and NCM 460 cells was first assessed using the CCK-8 assay [39]. The results demonstrated that even at a concentration as high as 200 µg/mL and after 24 h treatment, PRA@PCT exhibited no significant toxicity to either cell type (Fig. 2C and D).

To establish a cellular oxidative stress model, RAW 264.7 cells were stimulated with H_2_O_2_, and the appropriate concentration was determined using the CCK-8 assay. As shown in Fig. 2E, cell viability gradually decreased with increasing H_2_O_2_ concentrations. At a concentration of 200 µM H_2_O_2_, the cell viability was 68 ± 3%, indicating that the cells had undergone significant oxidative damage without irreversible cell death. This moderate damage level provides an ideal window for evaluating antioxidant intervention effects. Based on the experimental results and literature reports [40], 200 µM H_2_O_2_ was ultimately selected as the modeling concentration. The protective effect of different concentrations of PRA@PCT on H_2_O_2_-induced RAW 264.7 cells was further assessed using the CCK-8 assay. As illustrated in Fig. 2F, the viability of RAW 264.7 cells decreased to 67.3 ± 1.5% after stimulation with 200 µM H_2_O_2_. However, with increasing concentrations of PRA@PCT, cell viability significantly recovered; notably, at a concentration of 200 µg/mL PRA@PCT, cell viability reached 83 ± 2.6%, which was statistically significant compared to the model group.

Excessive ROS production is a hallmark of colitis-associated pathology [41–43]. Upon ROS stimulation, PRA@PCT dissociates and releases PRA, thereby alleviating cellular damage. To evaluate the protective effects of the drug on LPS stimulation, intracellular ROS levels in RAW 264.7 cells were detected using the fluorescent probe DCFH-DA. As displayed in Fig. 2G, the model group exhibited drastically elevated green fluorescence intensity, indicating massive intracellular accumulation of ROS. Treatment with SP, PRA, PRA@PCT, and SP@PRA@PCT all reduced fluorescence intensity to varying degrees. The SP group merely induced mild attenuation of ROS fluorescence, demonstrating that free SP possesses only moderate intrinsic ROS-scavenging capacity. PRA@PCT exerted superior ROS elimination efficiency compared with free PRA, which is attributed to its ROS-responsive targeted release property. Among all treatment groups, the SP@PRA@PCT group yielded the most remarkable reduction in fluorescence intensity, verifying prominent synergistic antioxidation between SP carrier and PRA@PCT. On the one hand, SP functions as a biological carrier to facilitate cellular internalization of PRA@PCT nanoparticles and increase intracellular PRA enrichment; on the other hand, natural antioxidant moieties released from SP synergistically eliminate surplus ROS together with responsively released PRA, jointly reinforcing the overall intracellular ROS-scavenging capability of the delivery system. Quantitative analysis of fluorescence signals further confirmed the potent ROS-scavenging capability of SP@PRA@PCT (Fig. S13A), and quantitative flow cytometry analysis was consistent with this trend (Fig. 2H). To further evaluate the mitigating effects of the SP@PRA@PCT on H_2_O_2_ stimulation, intracellular ROS levels were assessed. The results showed that the H_2_O_2_ group exhibited the highest ROS fluorescence intensity, while the SP@PRA@PCT group displayed the lowest fluorescence intensity (Figs. 2I and S13C), once again confirming the potent ROS scavenging capability of SP@PRA@PCT. This conclusion was further confirmed in NCM 460 cells (Figs. S13B and S13D). Calcein-AM/PI live/dead cell staining revealed that, compared with the H_2_O_2_-treated group, cell death progressively decreased in the PRA, PRA@PCT, and SP@PRA@PCT treatment groups, with the protective effect being most significantly pronounced in the SP@PRA@PCT group (Fig. 2J). Collectively, these findings demonstrate that SP@PRA@PCT effectively scavenges excessive intracellular ROS and protects cells from oxidative damage.

**Fig. 3:**
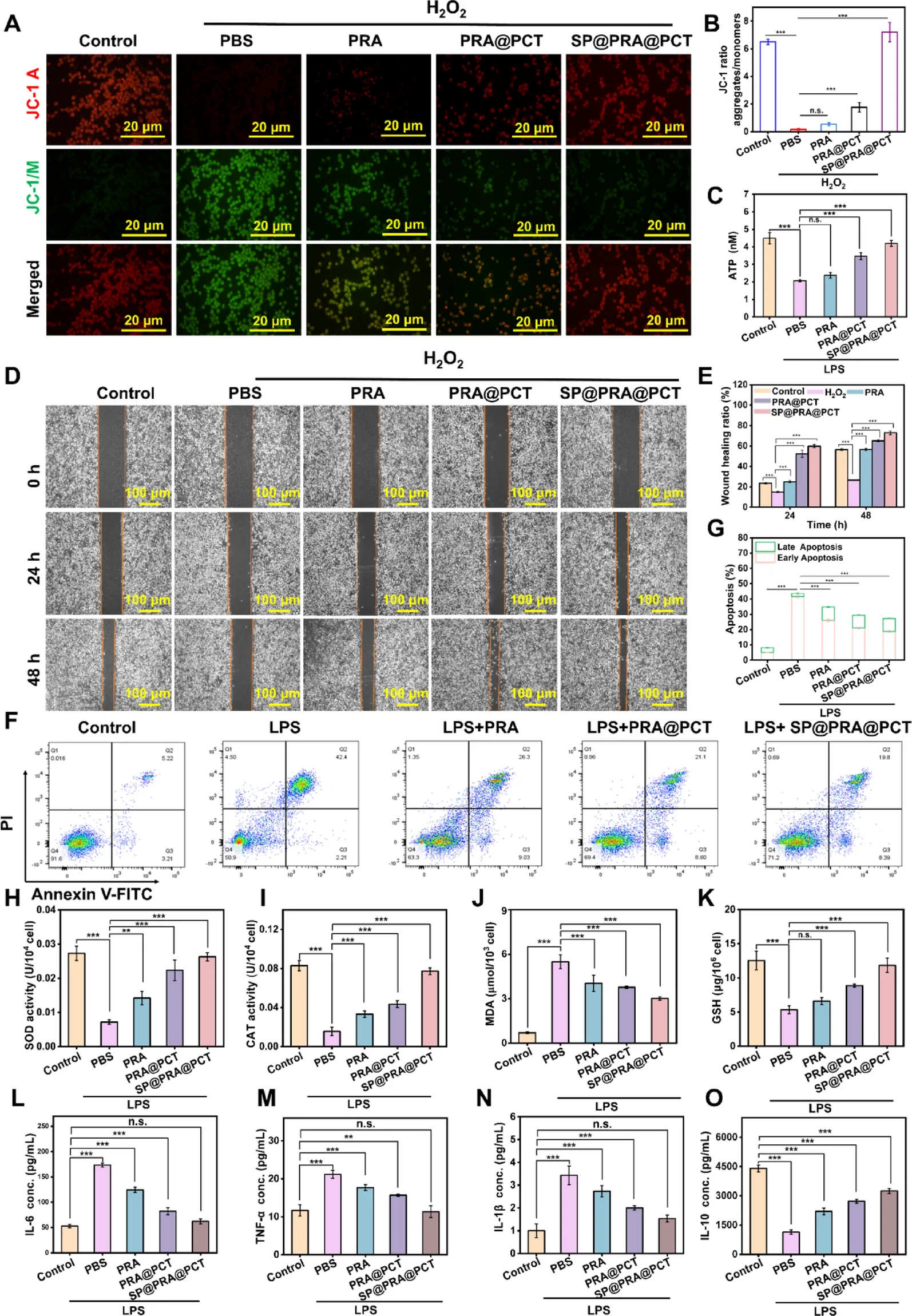
Analysis of mitochondrial function, cell migration, anti-apoptotic capacity, antioxidant activity, and anti-inflammatory effects of SP@PRA@PCT. (**A**) Fluorescence microscopic images of mitochondrial membrane potential in RAW 264.7 cells stained with JC-1 probe. (**B**) Quantitative analysis of JC-1 aggregate-to-monomer fluorescence intensity ratio. (**C**) Intracellular ATP content in RAW 264.7 cells treated with PRA, PRA@PCT, and SP@PRA@PCT. (**D**) Effects of PRA, PRA@PCT, and SP@PRA@PCT on scratch wound healing of NCM 460 cells. (**E**) Quantitative statistics of NCM 460 cell migration rate in the scratch wound healing assay. (**F**) Statistical analysis of the percentage of early and late apoptotic RAW 264.7 cells. (**G**) Flow cytometric analysis of cell apoptosis in RAW 264.7 cells using Annexin V/PI double staining. (**H-K**) Intracellular levels of SOD, CAT, MDA, and GSH in RAW 264.7 cells. (**L-O**) Secreted levels of IL-6, TNF-α, IL-1β, and IL-10 in RAW 264.7 cells. Data are mean ± SD (*n* = 3). *p < 0.05, **p < 0.01, ***p < 0.001, and n.s. = no significant

Oxidative stress is a primary inducer of mitochondrial dysfunction, leading to morphological changes and functional impairment of mitochondria, further impairing ATP synthesis [44]. Disruption of mitochondrial membrane potential further triggers irreversible apoptosis [45, 46]. Mitochondrial membrane potential (MMP) was detected using JC-1 fluorescent probe. The H_2_O_2_-treated group exhibited the strongest green fluorescence and the weakest red fluorescence, with a significantly reduced red/green fluorescence ratio, indicating MMP loss (Fig. 3A and B). Following drug treatment, this ratio was restored to varying degrees. Specifically, the PRA@PCT group showed superior efficacy compared to the free PRA group, attributable to the mitochondrial-targeting effect of CS-TPP, while the SP@PRA@PCT group exhibited the most pronounced recovery. A similar trend was validated in NCM 460 cells (Figures S13E and S13F). Furthermore, quantitative analysis of intracellular ATP levels revealed that SP@PRA@PCT treatment resulted in the most significant increase in ATP content (Fig. 3C). Collectively, these results demonstrate that SP@PRA@PCT ameliorates mitochondrial function by reducing ROS generation and alleviating energy metabolism disorders induced by oxidative stress.

Cell migration is crucial for tissue healing. As the primary barrier maintaining intestinal homeostasis, the colonic epithelium is susceptible to damage during UC progression. Therefore, promoting the migration of colonic epithelial cells is of significant importance for tissue repair [47, 48]. In this study, the effect of SP@PRA@PCT on the migration and repair capacity of NCM 460 intestinal epithelial cells was evaluated using a wound healing assay. As shown in Fig. 3D and E, all treatment groups promoted wound closure. After 24 h incubation, the cell migration rates for PRA, PRA@PCT, and SP@PRA@PCT were 25%, 52.4%, and 59.8%, respectively. By 48 h, the migration rate in the SP@PRA@PCT group further increased to 71.4%, which was significantly superior to both PRA and PRA@PCT groups, demonstrating its ability to effectively promote intestinal epithelial repair. On the one hand, sustained release of anthocyanins from SP@PRA@PCT effectively scavenges excess ROS and relieves migration suppression induced by oxidative stress. On the other hand, SP contains abundant phycocyanin and polysaccharides with inherent mucosa-protective and antioxidant activities, which directly facilitate epithelial migration and exert synergistic therapeutic effects with PRA@PCT. Collectively, these two advantages equip SP@PRA@PCT with superior capacity to repair damaged intestinal epithelium.

To further validate the cytoprotective capacity of SP@PRA@PCT, apoptosis in LPS-stimulated RAW 264.7 cells was quantitatively analyzed by flow cytometry. The results showed that LPS stimulation resulted in an apoptosis rate of 44.61% in RAW 264.7 cells, which was reduced to 28.19% following SP@PRA@PCT treatment, indicating its effectiveness in inhibiting oxidative stress-induced apoptosis (Fig. 3F and G). Additionally, the antioxidant effects of SP@PRA@PCT were further evaluated by assessing key oxidative stress indicators: superoxide dismutase (SOD) activity, catalase (CAT) activity, malondialdehyde (MDA) content, and glutathione (GSH) levels (Figs. 3H-K). Quantitative analysis revealed that treatment with PRA, PRA@PCT, and SP@PRA@PCT attenuated lipid peroxidation, enhanced CAT and SOD activities, and restored GSH levels. Concurrently, Elisa kit demonstrated that all treatment groups significantly reduced the expression of pro-inflammatory cytokines IL-6, TNF-α, and IL-1β, while upregulating the level of the anti-inflammatory cytokine IL-10 (Figs. 3L-O). In conclusion, SP@PRA@PCT ameliorates mitochondrial dysfunction, promotes cell migration, and inhibits apoptosis, demonstrating its promising potential as a candidate therapeutic material for the treatment of colitis.

### Intestinal distribution and retention of SP@PRA@PCT

**Fig. 4:**
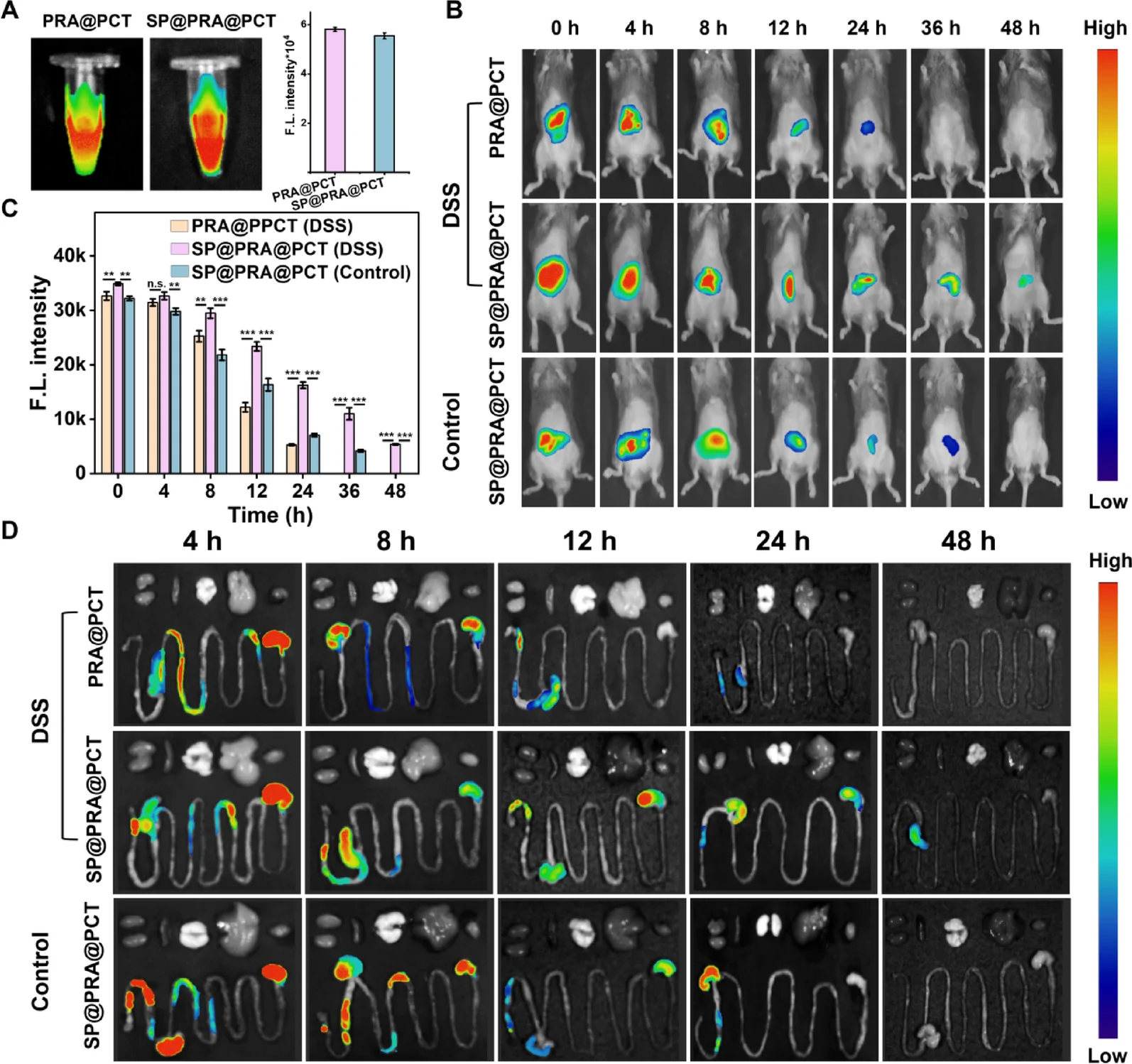
In vivo biodistribution study of SP@PRA@PCT. (**A**) In vitro fluorescence imaging and semi-quantitative analysis of fluorescence intensity for PRA@PCT and SP@PRA@PCT nanoparticles. (**B**) In vivo fluorescence distribution images of PRA@PCT and SP@PRA@PCT in normal and UC model mice at different time points post oral administration. (**C**) Semi-quantitative statistical analysis of fluorescence intensity for PRA@PCT and SP@PRA@PCT in normal and UC model mice. (**D**) Ex vivo fluorescence images of intestinal tissues from normal and UC model mice at different time points. Data are mean ± SD (*n* = 3). *p < 0.05, **p < 0.01, ***p < 0.001, and n.s. = no significant

To evaluate the retention property of SP@PRA@PCT in the intestinal tract, PRA@PCT was labeled with ICG-NHS fluorescent dye. Subsequently, fluorescence signals were monitored in both normal mice and UC model mice at predetermined time points after oral administration using an IVIS spectrum in vivo fluorescence imaging system. Figure 4A presents the fluorescence imaging and semi-quantitative analysis of fluorescence intensity for PRA@PCT and SP@PRA@PCT, demonstrating that the fluorescence intensities of both formulations were essentially consistent, indicating that any initial fluorescence differences between the drugs had a negligible impact on subsequent experiments.

In vivo imaging results (Fig. 4B and C) showed that in UC model mice, SP@PRA@PCT maintained a relatively high fluorescence intensity in the intestine for up to 48 h. In contrast, the fluorescence signal of PRA@PCT had substantially disappeared after 24 h. In the normal group administered with SP@PRA@PCT, the fluorescence intensity had largely vanished after 36 h. These results indicate that SP@PRA@PCT possesses superior targeted retention capability in the inflamed intestine. Fluorescence imaging of isolated intestinal tissues further validated this difference (Fig. 4D). In addition, no obvious fluorescence was detected in the major organs, including the heart, liver, spleen, lungs, and kidneys, suggesting that both formulations have good safety. In conclusion, the loading of PRA@PCT with SP prolongs its intestinal retention time, enabling sustained release of PRA, significantly improving its pharmacokinetic behavior, and enhancing the targeted therapeutic efficacy in inflamed intestinal segments.

### Evaluation of the therapeutic efficacy of SP@PRA@PCT in Colitis

**Fig. 5:**
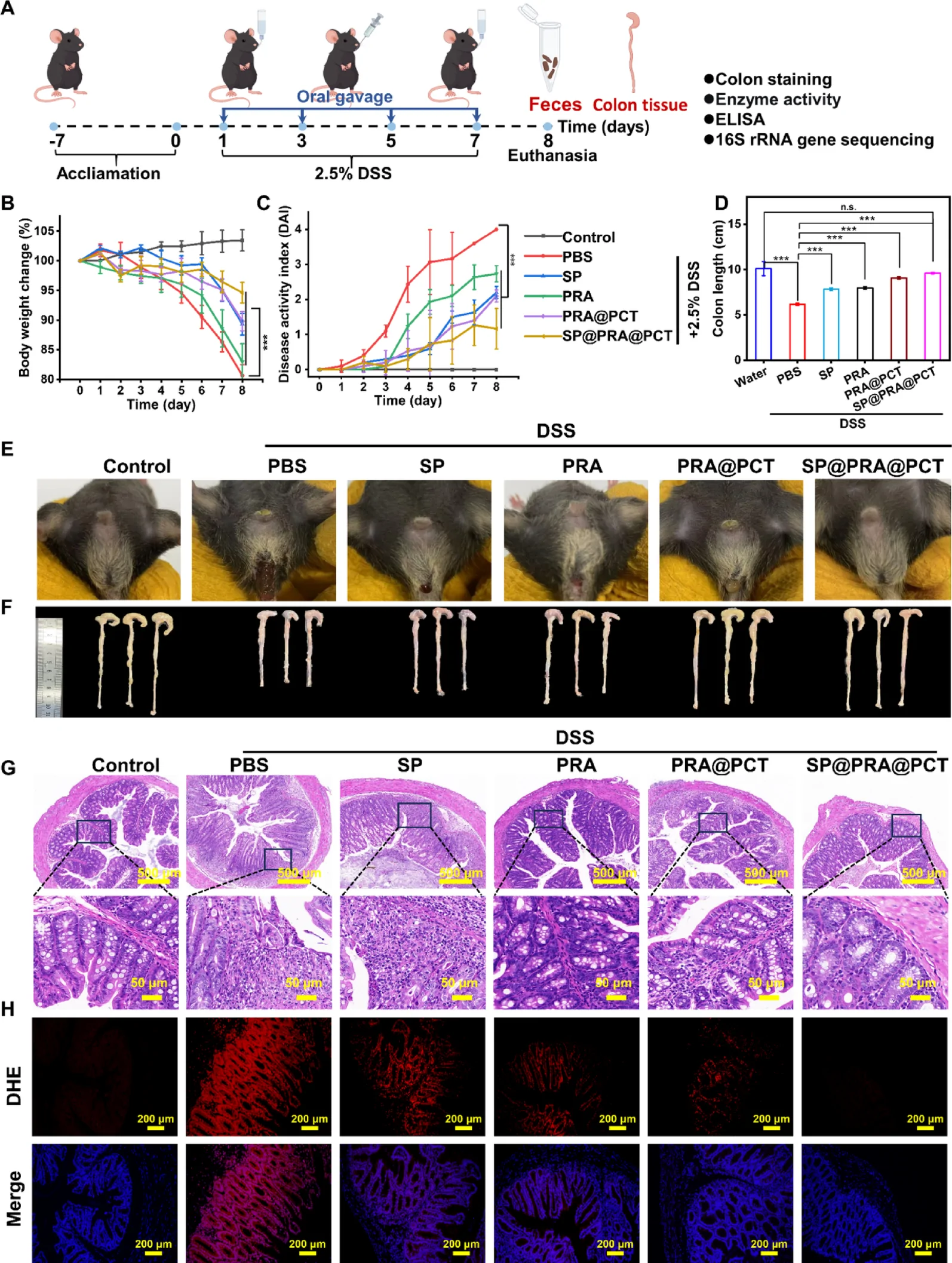
Therapeutic efficacy of SP@PRA@PCT in DSS-induced UC mice. (**A**) Schematic diagram of the animal experimental protocol. (**B**) Dynamic changes in body weight of mice in each group over the experimental period. (**C**) DAI scores of mice in each group during the intervention period. (**D**) Quantitative analysis of colon length in each treatment group. (**E**) Representative images of stool consistency and rectal bleeding in each group. (**F**) Representative photographs of colons isolated from each group. (**G**) Representative H&E-stained sections of colon tissues from each group. (**H**) Representative DHE-stained sections of colon tissues from each group. Data are mean ± SD (*n* = 6). *p < 0.05, **p < 0.01, and ***p < 0.001, and n.s. = no significant

Based on the potent antioxidant capacity, excellent intestinal retention, and precise drug release capability of SP@PRA@PCT, this study further evaluated its alleviating effects on UC mice and investigated its in vivo therapeutic potential. UC mouse models were established by administering 2.5% DSS in drinking water for 7 consecutive days. To systematically dissect the contribution of each functional module, clarify whether the superior efficacy stems from the intrinsic bioactivity of the components or the hierarchical delivery strategy, and further evaluate the clinical translational value of the formulation, multiple groups were set up in this study: Control group, DSS group, SP monotherapy group, free PRA group, free SP + PRA combination group, 5-ASA positive control group, PRA@PCT group, and SP@PRA@PCT group. As illustrated in Fig. 5A, mice in each treatment group received daily oral administration of corresponding formulations, while the Control and DSS groups were given an equal volume of PBS. As shown in Fig. 5B and Fig. S14A, mice in the DSS-induced group exhibited progressive and severe body weight loss, with approximately 20% reduction by day 7. All treatment groups inhibited body weight decline to varying degrees, with clear efficacy gradients among groups. Specifically, the free PRA group showed the weakest protective effect, with a final body weight loss rate of 17.3% on day 7. The SP monotherapy group achieved moderate relief, which was attributed to the inherent antioxidant and microbiota-regulating activities of SP. The free SP + PRA combination group presented a weight loss rate of 10.3%, showing a limited additive therapeutic effect compared with monotherapy groups, but still failed to achieve satisfactory protection. The PRA@PCT group (weight loss of 9.1%) exhibited better efficacy than the free drug combination, confirming the advantage of ROS-responsive delivery and mitochondrial targeting over free drug administration. Notably, the SP@PRA@PCT group showed the most pronounced inhibitory effect on body weight loss, with only 5.3% weight reduction at the end of modeling, which was significantly superior to both the free SP + PRA combination group and the 5-ASA positive control group.

The disease activity index (DAI) scores displayed a trend consistent with body weight changes (Fig. 5C, Fig. S14B). The DAI score of the DSS group increased sharply with disease progression, accompanied by severe diarrhea and hematochezia. Regarding hematochezia and stool consistency (Fig. 5E, Fig. S14D), the DSS group developed obvious bloody stools as early as day 4, while the SP and PRA monotherapy groups showed delayed onset until day 6. The 5-ASA group and free SP + PRA combination group showed milder symptoms than monotherapy groups. The PRA@PCT group exhibited further delayed onset and reduced severity, with only loose stools and occasional occult blood. In contrast, the SP@PRA@PCT group maintained the lowest DAI score throughout the observation period, with only mild soft stools and no visible bloody stools during the whole experiment, indicating that disease progression was most effectively delayed. Colon length serves as a key indicator for evaluating therapeutic efficacy in UC [49]. As shown in Fig. 5D and F and Figs. S14C, S14E, compared with the healthy control group (9.7 ± 0.3 cm), the DSS-induced colitis group exhibited a marked reduction in colon length to 6.1 ± 0.2 cm. Although all treatment groups inhibited colon shortening to varying degrees, the SP@PRA@PCT group demonstrated the most pronounced effect, restoring colon length to 9.6 ± 0.1 cm, which approached that of normal controls and was significantly superior to both the free drug combination and the 5-ASA group.

The results revealed that the SP monotherapy group and free PRA group exhibited moderate anti-inflammatory and mucosal repair effects, which stem from the inherent bioactivities of SP and PRA. Nevertheless, such single-agent administration and even their simple combination failed to achieve satisfactory therapeutic outcomes, indicating that the intrinsic pharmacological activity of the components is not the core driver of the high efficacy of SP@PRA@PCT. After loading PRA@PCT nanoparticles onto SP, the delivery system achieved dual-level functional synergies. On one hand, the helical microstructure and natural mucoadhesive property of SP prolonged the intestinal residence of the formulation at inflamed colonic sites, enabling sustained local enrichment and on-demand release of the core bioactive PRA. On the other hand, the intrinsic antioxidant and gut microbiota-modulating capacities of SP exerted synergistic therapeutic effects with the mitochondrial-targeted ROS scavenging function of PRA. Additional comparative validation demonstrated that the SP@PRA@PCT group was significantly more effective than the PRA@PCT group, directly confirming the irreplaceable role of the microalgae-based delivery platform in improving local drug bioavailability. Meanwhile, its therapeutic performance was markedly better than that of the equivalent-dose free SP+PRA combination group, firmly verifying that the formulation’s therapeutic advantage stems from the hierarchical delivery strategy of intestinal adhesion and enrichment, ROS-responsive stepwise release, and mitochondrial targeting. Importantly, the efficacy of SP@PRA@PCT outperformed the clinical first-line drug 5-ASA in multiple core indicators, suggesting its good clinical translational potential.

### Restoration of intestinal barrier function and in vivo anti-inflammatory/antioxidant effects by SP@PRA@PCT

H&E staining was performed to evaluate the histopathological damage of colon tissues in each group. As shown in Fig. 5G and Fig. S14F, the DSS-induced group exhibited extensive mucosal necrosis, severe inflammatory cell infiltration, massive crypt loss, and ulcer formation in colon tissues, indicating severe destruction of intestinal barrier structure. All treatment groups mitigated the degree of colonic histopathological injury to varying extents. Specifically, the free PRA group showed only limited improvement, with still widespread inflammatory infiltration and crypt destruction. The SP monotherapy group and free SP + PRA combination group showed reduced ulcer area and inflammatory cell infiltration to a certain extent, but there were still focal crypt structure disruption, disorganized gland arrangement, and moderate inflammatory cell infiltration in the lamina propria, indicating limited mucosal barrier repair effect of free active components. The 5-ASA positive control group showed alleviated mucosal injury, but partial crypt atrophy and inflammatory infiltration still existed. The PRA@PCT group showed better tissue repair effect, with relatively intact epithelial structure and reduced inflammatory infiltration. Notably, the SP@PRA@PCT treatment group exhibited the most significant protective effect on colon tissue: the colonic mucosal epithelium was continuous and intact, the crypt structure was regular and clear, goblet cells were abundant, and inflammatory cell infiltration was minimal, which was close to the histological state of the normal control group. Colonic histological injury scores (Fig. S15) provided further quantitative corroboration of the aforementioned trend, demonstrating that SP@PRA@PCT effectively ameliorates colonic mucosal damage and restores intestinal barrier integrity. Its histological repair efficacy was significantly superior to that of both the free drug combination and the first-line clinical drug 5-ASA.

**Fig. 6:**
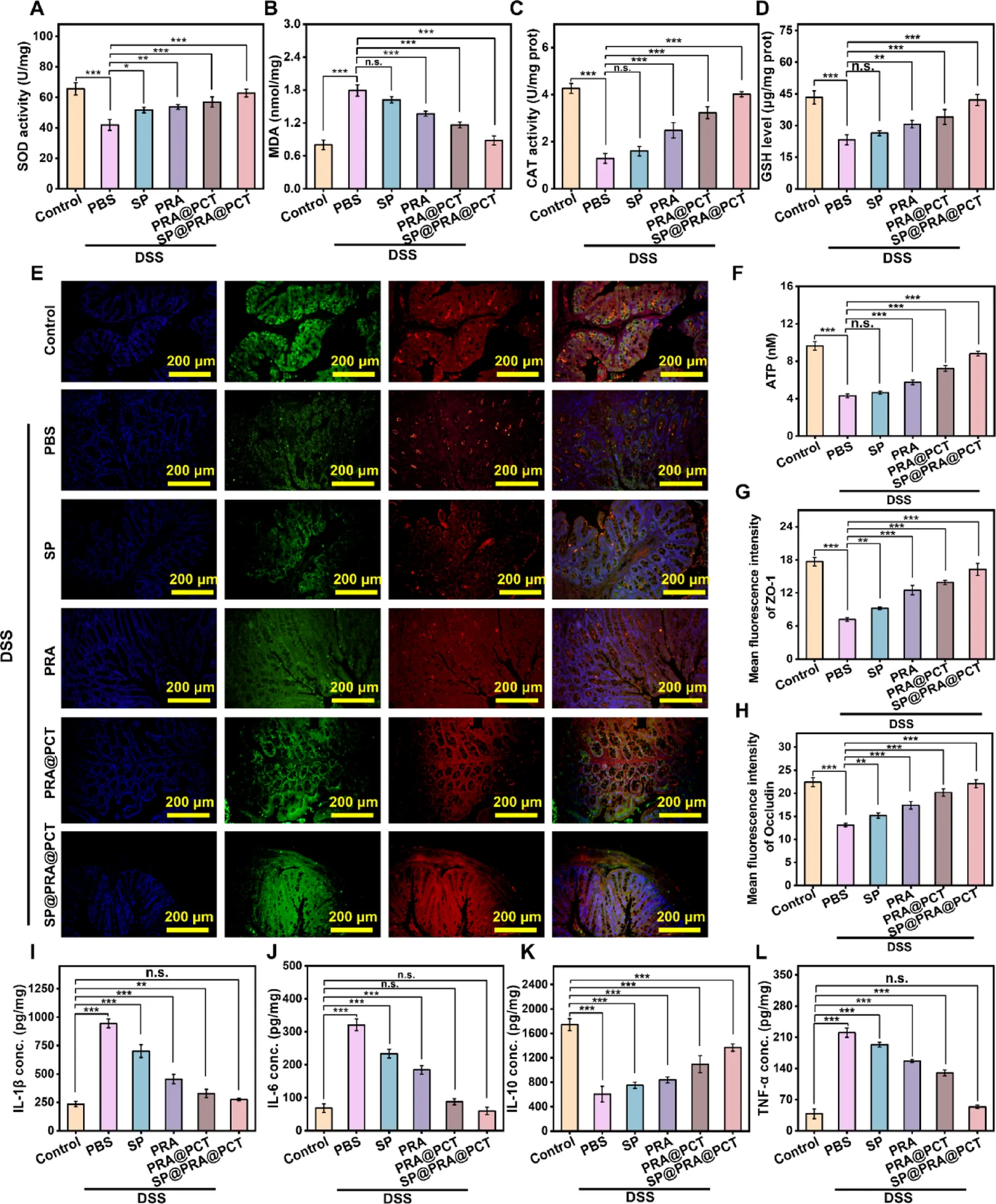
Restoration of intestinal barrier function and in vivo anti-inflammatory/antioxidant effects by SP@PRA@PCT. The activity of (**A**) SOD and (**B**) CAT in colon tissues of mice in each group. The content of (**C**) MDA, (**D**) GSH and (**F**) ATP in colon tissues of mice in each group. (b) Representative immunofluorescence staining images of ZO-1 and Occludin in mouse colon tissues. Semi-quantitative analysis of immunofluorescence intensity for (G) ZO-1 and (H) Occludin in mouse colon tissues. Tissue levels of (I) IL-1β, (J) IL-6, (K) IL-10, and (L) TNF-α in colon tissues in each group. Data are mean ± SD (*n* = 6). *p < 0.05, **p < 0.01, ***p < 0.001, and n.s. = no significant

The progression of UC is often accompanied by exacerbated oxidative stress, primarily characterized by excessive mitochondrial ROS production, reflecting an imbalance between oxidants and the antioxidant system. Therefore, scavenging excess ROS has been regarded as a crucial strategy for UC alleviation [50, 51]. To evaluate the in vivo antioxidant capacity of SP@PRA@PCT, DHE staining was performed on colonic tissues, and the levels of ATP, MDA, GSH, as well as the activities of SOD and CAT enzymes, were measured. DHE fluorescence staining showed that ROS levels were significantly elevated in the colon of the DSS group, whereas SP@PRA@PCT intervention markedly reduced ROS levels, demonstrating its potent ROS-scavenging capacity (Fig. 5H). Moreover, as shown in Figs. 6A-D, the DSS group displayed decreased SOD and CAT activities, reduced GSH content, and increased MDA levels. All treatment groups reversed these alterations to varying degrees, with SP@PRA@PCT exhibiting the most pronounced effects by enhancing CAT and SOD activities, restoring GSH levels, and reducing MDA content. Further analysis revealed that the SP@PRA@PCT group exhibited the most substantial increase in ATP levels within colonic tissues (Fig. 6F), suggesting that SP@PRA@PCT alleviates colonic tissue damage by targeting mitochondria and inhibiting ROS generation. Collectively, these results demonstrate that SP@PRA@PCT possesses robust antioxidant capacity in vivo and effectively alleviates UC-associated oxidative stress.

Intestinal barrier dysfunction is a critical event in UC pathogenesis, with tight junction proteins such as ZO-1 and Occludin playing essential roles in maintaining intestinal barrier integrity [52, 53]. Immunofluorescence staining results demonstrated that, compared with the normal group, the expression of ZO-1 and Occludin in the DSS group was markedly decreased and exhibited disrupted distribution (Fig. 6E). All treatment groups restored the expression of these proteins to some extent, with the SP@PRA@PCT group showing levels closest to those of the normal group. These findings indicate that SP@PRA@PCT effectively repairs the damaged intestinal epithelial barrier and preserves intestinal barrier integrity.

UC development is frequently associated with dysregulated cytokine secretion, characterized by elevated pro-inflammatory cytokine levels and reduced anti-inflammatory cytokine levels [54–56]. Accordingly, the expression levels of pro-inflammatory markers (IL-6, TNF-α, and IL-1β) and the anti-inflammatory marker (IL-10) in mouse colon tissues were assessed using Elisa kits. As presented in Figs. 6I-L, the DSS group exhibited significantly increased levels of pro-inflammatory cytokines IL-6, TNF-α, and IL-1β), along with a marked decrease in the anti-inflammatory cytokine IL-10. Although all treatment groups reversed this dysregulation to varying degrees, the modulatory effect of SP@PRA@PCT was the most pronounced. In summary, SP@PRA@PCT effectively regulates cytokine expression and alleviates UC-associated inflammation.

### Regulatory effect of SP@PRA@PCT on gut microbiota in UC mice

**Fig. 7:**
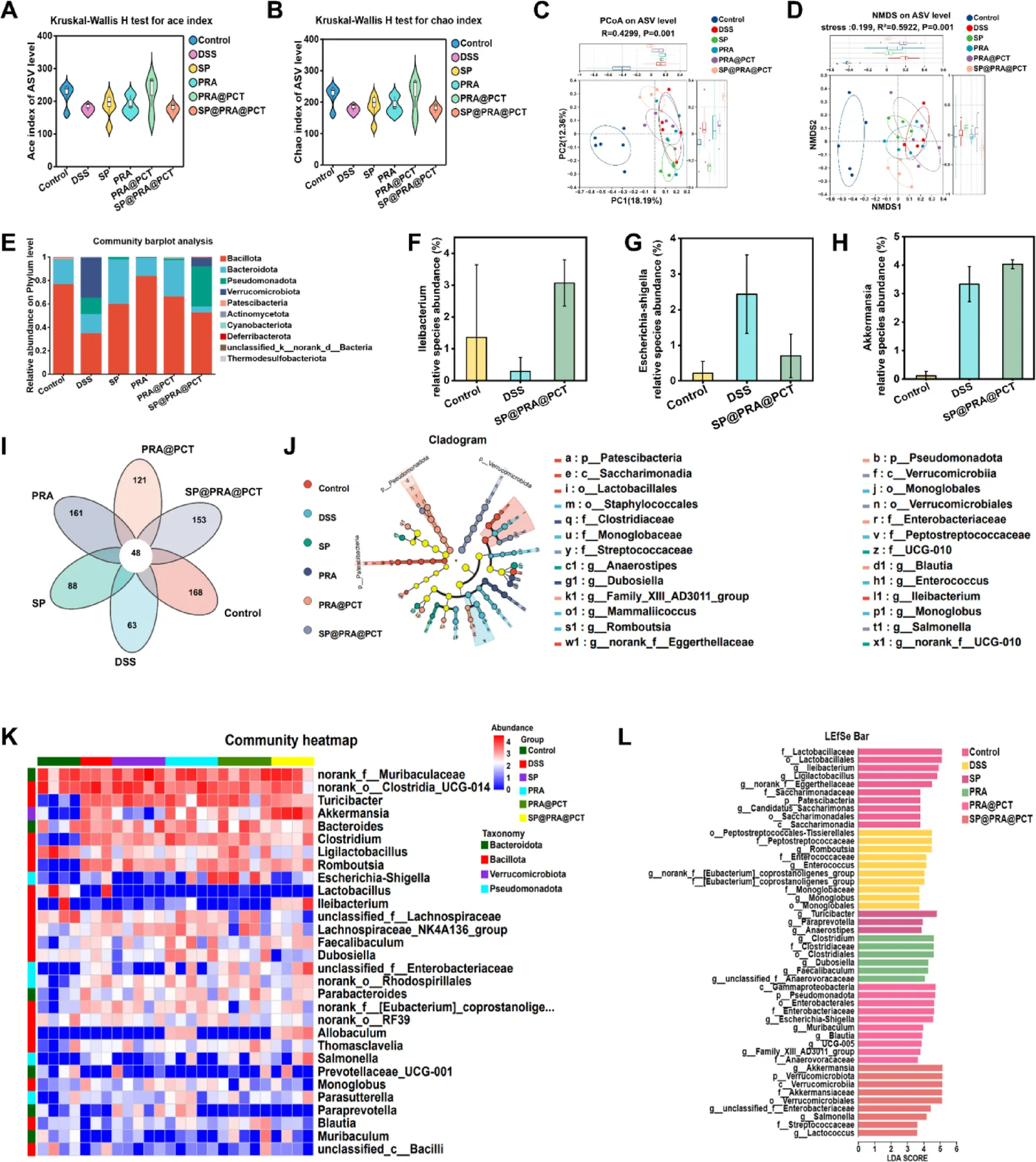
Modulation of gut microbiota by SP@PRA@PCT in DSS-induced UC mice. (**A**,** B**) Microbial richness indices, including ACE and Chao1. (**C**) PCoA and (**D**) NMDS plots illustrating gut microbial β-diversity. (**E**) Relative abundance of gut microbiota at the phylum level among different treatment groups. (**F-H**) Relative abundance of Akkermansia, Escherichia-Shigella, and Ileibacterium across different taxonomic levels. (**I**) Venn diagram depicting the shared and unique species richness among groups. (**J**) Cladogram derived from linear discriminant analysis effect size (LEfSe) analysis. (**K**) Heatmap showing the relative abundance of gut microbiota across all groups. (**L**) Linear discriminant analysis (LDA) scores for discriminating microbial taxa with significant differences. Data are mean ± SD (Normal group: *n* = 6, DSS group: *n* = 5, SP group: *n* = 5, PRA group: *n* = 5, PRA@PCT group: *n* = 5, SP@PRA@PCT group: *n* = 4). *p < 0.05, **p < 0.01, ***p < 0.001, and n.s. = not significant

Numerous studies have demonstrated that maintaining the balance of gut microbiota is crucial for the treatment of UC [57–59]. To investigate the modulatory effect of SP@PRA@PCT on the gut microbiota, this study employed 16 S rRNA gene sequencing technology to perform gene amplification and sequencing on fresh fecal samples. Violin plots of the Alpha diversity indices (Ace and Chao) revealed that, while microbial richness and alpha diversity showed partial recovery following treatment, the magnitude of this increase was modest and did not indicate a clear restoration of microbial richness (Fig. 7A and B). Nevertheless, it is important to note that increased microbial diversity in inflammatory bowel disease does not consistently correlate with improved gut health, as such increases may arise from the expansion of pathogenic taxa [60]. Beta diversity analysis demonstrated that the microbial distribution in the SP@PRA@PCT group was positioned between the normal and model groups and exhibited a pronounced shift toward the normal group, indicating that this treatment contributed to restoring a relatively normal microbial community structure (Fig. 7C and D).

Further analysis of microbial composition changes at the phylum and genus levels was performed (Fig. 7E). The results showed that under inflammatory conditions, the abundance of Escherichia-Shigella, a pathogenic genus within the Proteobacteria phylum, was significantly increased. Following SP@PRA@PCT treatment, the abundance of beneficial bacteria, including Akkermansia (belonging to the phylum Verrucomicrobiota) and Ileibacterium (a genus belonging to Bacillota that has been reported in previous literature to be associated with the metabolism of SCFAs including butyrate) [54], was markedly elevated. In contrast, the abundance of Escherichia-Shigella was reduced (Fig. 7F and G, and 7H). These changes are conducive to enhancing intestinal barrier function and ameliorating metabolic disorders. Venn diagram analysis revealed that the control, DSS, and SP@PRA@PCT-treated groups expressed 168, 63, and 153 species-specific amplicon sequence variants, respectively, confirming that SP@PRA@PCT could restore the number of species diminished by inflammation (Fig. 7I).

To further investigate the characteristics of microbial changes, Linear Discriminant Analysis (LDA) and LEfSe cladogram analysis were performed (Fig. 7J and L). The LDA plot indicated that the dominant microbiota in the SP@PRA@PCT group included Akkermansiaceae, Verrucomicrobiota (phylum), and Akkermansia (genus) within Verrucomicrobiota. Additionally, a heatmap of gut microbial abundance at the genus level illustrated the trends in key microbial populations (Fig. 7K). Heatmap analysis further illustrated the correlations among the gut microbiome, inflammation‑related factors, and histopathological scores (Fig. S16). Ileibacterium was positively correlated with the pro‑inflammatory cytokines IL‑6, TNF‑α, and IL‑1β, as well as with the DAI score and histopathological score. In contrast, Escherichia‑Shigella was negatively correlated with the anti‑inflammatory cytokine IL‑10 and colon length. These findings collectively demonstrate that SP@PRA@PCT regulates gut microbiota dysbiosis by increasing the abundance of beneficial bacteria such as Ileibacterium, while inhibiting pathogenic bacteria, including Escherichia-Shigella, thereby alleviating intestinal inflammation and promoting gut microecological health.

### In vivo safety evaluation of SP@PRA@PCT

**Fig. 8:**
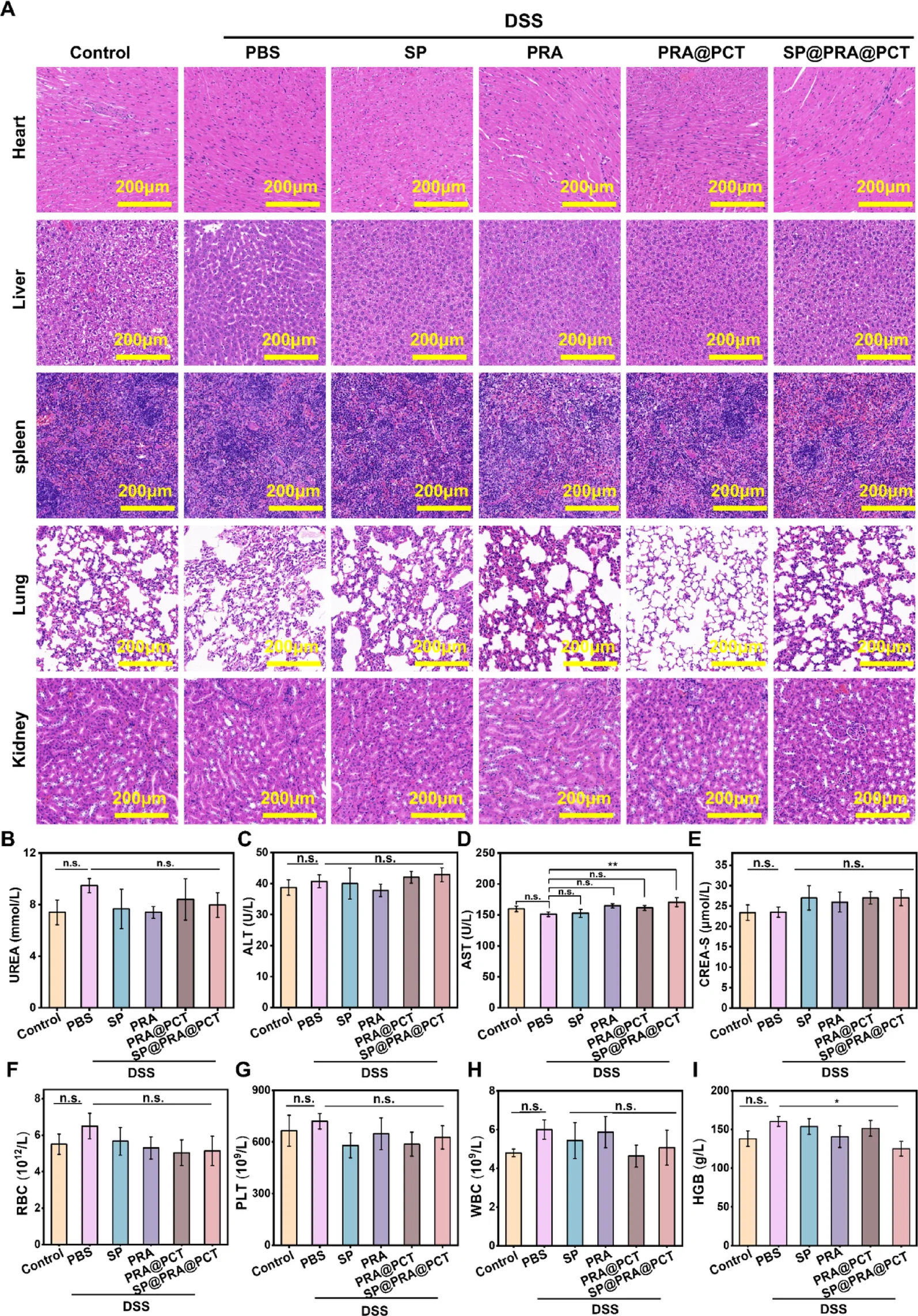
In vivo safety study. (**A**) Representative H&E staining images of major organs from mice. (**B-E**) Serum levels of UREA (**B**), ALT (**C**), AST (**D**), and CREA-S (**E**) in mice from different groups. (**F-I**) Blood cell counts of RBC (**F**), PLT (**G**), WBC (**H**), and HGB (**I**) in mice from different groups. Data are mean ± SD (*n* = 6). *p < 0.05, **p < 0.01, ***p < 0.001, and n.s. indicating no significant difference

To further evaluate the in vivo biosafety of SP@PRA@PCT, histopathological analysis of major organs from mice after treatment was performed, along with assessment of blood biochemical and routine parameters. H&E staining of major organs (heart, liver, spleen, lungs, and kidneys) is presented in Fig. 8A. In all experimental groups, heart tissues exhibited typical fibrous structural characteristics, with cardiomyocytes arranged compactly and interconnected, forming an interlaced reticular distribution pattern. Liver tissues displayed intact morphological structure, with regularly arranged hepatic cell cords and clearly discernible hepatic lobules. Lung tissues showed clear alveolar morphology and structure, with no observable abnormal pathological changes such as inflammatory cell infiltration or pulmonary interstitial hyperplasia. In kidney tissues, no abnormalities were observed in the morphological structure of glomeruli, renal tubules, or renal cortex, nor were there any pathological damages such as inflammatory cell infiltration or cell necrosis. These results indicate that treatment with SP, PRA, PRA@PCT, and SP@PRA@PCT did not induce significant pathological changes or inflammatory damage in the major organs of mice.

Blood biochemical analysis revealed that alanine aminotransferase (ALT) and aspartate aminotransferase (AST), which reflect liver function, as well as creatinine (CREA-S) and urea (UREA), which reflect kidney function, were all within normal physiological ranges, indicating that SP@PRA@PCT did not cause significant liver or kidney functional damage (Fig. 8B-E). Furthermore, complete blood count analysis demonstrated that white blood cell (WBC), red blood cell (RBC), hemoglobin (HGB), and platelet (PLT) counts in all groups were also within normal reference ranges, suggesting that this formulation had no significant effects on the hematopoietic system or immune cells (Fig. 8F-I).

In conclusion, SP@PRA@PCT did not induce histopathological changes in major organs, nor did it significantly affect liver and kidney function or blood routine parameters in mice, demonstrating excellent biocompatibility and in vivo biosafety. These findings provide an important safety basis for its further application in the treatment of ulcerative colitis.

## Conclusion

In this study, we successfully constructed a microalgae-loaded ROS-responsive PRA nano-delivery system (SP@PRA@PCT) for targeted therapy of UC. In this system, PRA extracted from purple carrots was encapsulated within the ROS-responsive polymer PPADT to form core nanoparticles (PRA@PCT). Subsequent modification with CS-TPP conferred mitochondrial targeting capability and positive surface charge, enabling efficient loading onto the surface of SP. In vitro studies demonstrated that PRA@PCT exhibited excellent stability in simulated gastrointestinal fluids while undergoing rapid degradation and PRA release specifically in ROS-rich colon environments, accompanied by superior free radical scavenging capacity. In cellular oxidative stress models, PRA released from PRA@PCT effectively eliminated intracellular ROS, restored mitochondrial membrane potential, and inhibited oxidative stress-induced apoptosis. Following oral administration in a UC mouse model, SP@PRA@PCT achieved prolonged retention (up to 48 h) in the inflamed intestine, facilitating colon-specific delivery of PRA and exerting therapeutic effects, including antioxidant, anti-inflammatory, and gut microbiota modulation. Notably, PRA@PCT exhibited no significant toxicity in either in vitro or in vivo evaluations, demonstrating favorable biocompatibility and clinical translational potential. Collectively, this oral microalgae-based ROS-responsive nano-delivery system represents a promising therapeutic strategy for UC, offering broad application prospects in the precision treatment of ROS-associated inflammatory bowel diseases.

## Experimental section

Experimental procedures encompassing materials, preparation and characterization of SP@PRA@PCT, measurement of ROS response, and biological assessments under both in vitro and in vivo conditions are systematically documented in the Supporting Information.

C57BL/6 male mice (6–8 weeks) were purchased from the Animal Experiment Center of Shanxi Medical University, and all procedures were conducted in compliance with the regulations of the Ethics Committee of Shanxi Medical University.

All data are presented as mean ± standard deviation (SD). Statistical significance was determined by one-way analysis of variance (ANOVA) using SPSS software, with significance levels denoted as ^∗^*p* < 0.05, ^∗∗^ *p* < 0.01, ^∗∗∗^ *p* < 0.001, and n.s. indicating no significant difference.

## Supplementary Information

Supplementary Material 1

## Data Availability

No datasets were generated or analysed during the current study.
