# Supplementary material for: Oral microalgae-ROS-responsive hierarchical targeting delivery system for precision therapy of ulcerative colitis
Source: J Nanobiotechnology. 2026 Aug 6;24:1016. doi: 10.1186/s12951-026-04890-5 (PMC13729452; doi:10.1186/s12951-026-04890-5)
Supplement: Supplementary file 1 [file 12951_2026_4890_MOESM1_ESM.docx]

**Oral microalgae-ROS-responsive hierarchical targeting delivery system for precision therapy of ulcerative colitis**

Ru Feng ^a,#^, Yongle Zhao ^a,#^, Xiang Sun ^b,#^, Luke Ge ^a^, Pengqi Zhu^a^, Wanying Zhang ^a^, Caifang Gao^a,*^,Jinghua Sun ^c,*^, and Ruiping Zhang ^b,*^

*a Shanxi Bethune Hospital, Shanxi Academy of Medical Sciences, Third Hospital of Shanxi Medical University, Tongji Shanxi Hospital, Taiyuan, 030032, China*

*b Department of Radiology, Fifth Hospital of Shanxi Medical University, Shanxi Provincial People’s Hospital, Taiyuan 030000, China*

*c First Hospital of Shanxi Medical University, Taiyuan 030001, China*

\# Ru Feng, Yongle Zhao, and Xiang Sun contributed equally to this study.

*Correspondence: Caifang Gao, gaocaifang@sxbqeh.com.cn; Jinghua Sun, sunjh0000@163.com; Ruiping Zhang, zrp_7142@sxmu.edu.cn.

**4. Materials and Methods**

4.1 Materials

Purple carrots were purchased from Guanxian Woze Agriculture Technology Co.Ltd. N-Hydroxysulfosuccinimide (NHS), 1-ethyl-3-(3 dimethylaminopropyl) carbodiimide (EDC), oligomericchitosan, and (4-Carboxybutyl) triphenylphosphonium bromide (TPP) were obtained from Shanghai Macklin Biochemical Co. Ltd. (Shanghai, China). ICG-NHS was supplied by Xi'an Dianhua Biotechnology Co.Ltd. 2,2-Dimethoxypropane (99%) and sodium cholate (98%) were purchased from Aladdin Reagent Co. Ltd. (Shanghai, China). 1,4-Benzenedimethanethiol (95%) was acquired from Shanghai Energy Chemical Co. Ltd. (China). p-Toluenesulfonic acid (98.5%) was purchased from Sigma-Aldrich. Hydrochloric acid (37%), dichloromethane, toluene, acetone, ethyl acetate, and n-hexane were obtained from Sinopharm Chemical Reagent Beijing Co. Ltd. Macroporous resin (LSA-10) was supplied by Beijing Zhongran Xusheng Technology Development Co. Ltd. Dextran sulfate sodium (DSS) was purchased from Shanghai Bide Pharmaceutical Technology Co. Ltd. Reactive oxygen species (ROS) assay kit and mitochondrial membrane potential assay kit (JC-1) were procured from Beijing Solarbio Science & Technology Co. Ltd. Oxidative stress-related assay kits (for SOD, CAT, MDA, and GSH) were also obtained from Beijing Solarbio Science & Technology Co. Ltd. Cytokine enzyme-linked immunosorbent assay (Elisa) kits (for TNF-α, IL-1β, IL-6, and IL-10) were purchased from ELISA MAX Deluxe Set, Biolegend (USA).

4.2 Extraction, Purification and Characterization of Purple Radish Anthocyanins (PRA)

Fresh purple carrots were shredded, dried at 60 °C, and ground into a fine powder. PRA were extracted twice using a hydrochloric acid-75% ethanol solution (pH 2-3) as the extraction solvent, with a solid-to-liquid ratio of 1:5 (w/v). Each extraction was conducted under ultrasonication (450 W) at 60 °C for 30 min. After filtration to remove insoluble residues, the combined extracts were loaded onto a pre-activated macroporous resin (LSA-10) column and subjected to static adsorption for 20 h. Impurities such as sugars and interfering pigments were eluted with distilled water, followed by elution of the target PRA using a 60% ethanol solution. The anthocyanin-containing eluate was concentrated by rotary evaporation at 50 °C and subsequently vacuum-dried at 55 °C for 36 h to obtain purified PRA powder, which was stored at 4 °C until further use.

Qualitative and quantitative analyses of the extracted PRA were performed using high-performance liquid chromatography (HPLC). Briefly, 1.0 mg of the purified PRA powder was dissolved in 5 mL of distilled water, filtered through a 0.45 μm syringe filter, and degassed by ultrasonication. A 20 μL aliquot of the sample was injected into the HPLC system for analysis. Chromatographic separation was carried out on an Alphasil VC-C18 column (5 μm, 4.6×150 mm) maintained at 40 °C, with a detection wavelength of 530 nm. The mobile phase consisted of acetonitrile (solvent A) and a mixed solution of phosphoric acid, formic acid, acetonitrile, and distilled water (1:10:5:84, v/v/v/v) (solvent B), delivered at a constant flow rate of 1.0 mL/min. The gradient elution program was set as follows: 0 min, 1% A / 99% B; 10 min, 12% A / 88% B; 20 min, 15% A / 85% B; 25 min, 32% A / 68% B; 30 min, 1% A / 99% B. The morphology of the extracted PRA particles was observed via transmission electron microscopy (TEM).

To validate the reliability of the HPLC method, the linearity, recovery, and reproducibility were systematically evaluated. A series of mixed standard solutions at different concentrations were prepared, and calibration curves were constructed by linear regression of peak area (y) against concentration (x, μg/mL). Recovery tests were performed using the standard addition method, in which standard solutions at six concentration levels were spiked into blank samples, and the recoveries were calculated accordingly. Reproducibility was assessed by repeatedly analyzing the same batch of samples six times under identical chromatographic conditions, and the RSD values of the contents of each component were calculated to evaluate the precision of the method.

4.3 Synthesis and Characterization of Poly(aryloxy-pentanedione thioketal) (PPADT)

PPADT was synthesized via a condensation polymerization reaction as follows: 1,4-benzenedimethanethiol (0.68 g) and 2,2-dimethoxypropane (492 µL) were dissolved in 25 mL of anhydrous toluene. The reaction mixture was heated to 105 °C under continuous magnetic stirring. A catalyst solution, prepared by dissolving *p*-toluenesulfonic acid (4.57 mg) in ethyl acetate (100 µL), was then added dropwise to the reaction system. After 1 h of reaction at 105 °C, an additional solution of 2,2-dimethoxypropane (492 µL) in toluene (24 mL) was added via a syringe pump at a constant rate of 2 mL/h over 12 h. The reaction mixture was stirred overnight, and after the reaction was completed, the polymer was isolated by precipitation in cold n-hexane. The final product was obtained after vacuum drying.

4.4 Synthesis and Characterization of Chitosan-Triphenylphosphonium (CS-TPP)

(4-Carboxybutyl) triphenylphosphonium bromide (TPP) was dispersed in 25 mL of 2-(N-morpholino) ethanesulfonic acid (MES) buffer (0.1 M, pH 5.0). 1-Ethyl-3-(3-dimethylaminopropyl) carbodiimide (EDC, 400 mg) and N-hydroxysulfosuccinimide (NHS, 200 mg) were then added to the dispersion. The mixture was stirred at room temperature for 5 h to activate the carboxyl groups of TPP. Separately, chitosan (200 mg) was dispersed in 25 mL of a 0.1% (w/v) acetic acid solution (pH adjusted to 5.0). This chitosan dispersion was subsequently added to the activated TPP solution. The reaction was allowed to proceed at room temperature with stirring at 600 rpm for 10 h. The resulting product was purified by dialysis against distilled water using a dialysis membrane (molecular weight cut off: 500 Da) for 2-3 days, followed by lyophilization to obtain the final CS-TPP conjugate. The structure of CS-TPP was confirmed by ^1^H NMR spectroscopy using a nuclear magnetic resonance spectrometer (Bruker, 400 MHz, Germany).

4.5 Synthesis of PPADT-Coated PRA Nanoparticles

PRA@PPADT nanoparticles were prepared using an emulsion and solvent evaporation method. First, sodium cholate hydrate (5 mg) was added to 500 μL of a PRA solution (10 mg/mL) to obtain the aqueous phase. This aqueous phase was then added to 5 mL of dichloromethane containing PPADT (100 mg). For Nile Red-labeled PRA@PCT, 1 mg of Nile Red was dissolved in this solution. The mixture was emulsified by probe ultrasonication at 200 W with a 50% duty cycle for 30 seconds. Subsequently, the resulting primary emulsion was added to 10 mL of sodium cholate hydrate solution (10 mg/mL) and sonicated again for 30 seconds. After that, the system was transferred into 50 mL of sodium cholate hydrate solution (5 mg/mL) and stirred at room temperature for 4 h. The mixture was then transferred into a dialysis membrane with a molecular weight cutoff of 10 kDa and dialyzed against deionized water for 48 h to obtain PRA@PPADT nanoparticles. Finally, the Chitosan-TPP solution (0.5 mL, 1 mg/mL) was added to the PRA@PPADT, and the reaction was stirred for an additional 3 h to obtain the final products: PRA@PCT, FITC-PRA@PCT, and FITC/NileRed-PRA@PCT. To obtain ICG-labeled PRA, PRA was modified with NH_2_-PEG-NH_2_ according to a previous study, followed by the addition of ICG-NHS fluorescent dye and stirring for 15 min. Free dye was removed by ultrafiltration concentration. ICG-labeled PRA@PCT and SP@PRA@PCT were then prepared using the same method as for the material preparation. The morphology of the PRA@PCT solution before and after incubation with hydrogen peroxide (H_2_O_2_) solution was observed using a transmission electron microscope. The hydrodynamic diameter and zeta potential of the materials were determined using a Malvern Zetasizer (Malvern Instruments). The infrared absorption spectra of PPADT, PRA@PPADT, and PRA@PPADT after incubation with 0.1 mM H_2_O_2_ solution were measured using a Fourier transform infrared spectrometer (Bruker, Germany).

4.6 Synthesis and Characterization of the SP@PRA@PCT

Spirulina platensis (SP) were cultivated in Zarrouk medium under constant illumination at 30 ^o^C. The cultured SP was washed three times with PBS and subsequently lyophilized. The SP samples were imaged using scanning electron microscopy (SEM, model: JSM‑IT800, accelerating voltage: 5 kV) at a magnification of 200×, and randomly collected images from different fields of view. To obtain statistically meaningful results, the particle sizes of SP particles were measured using ImageJ, and statistical analysis was performed on all measured data. To assemble SP@PRA@PCT, the microalgae were stirred with PRA@PCT (40:1) at room temperature for 15 min to obtain SP@PRA@PCT. The structural characteristics of SP and SP@PRA@PCT were observed using SEM, TEM, and confocal laser scanning microscopy (CLSM).

4.7 Encapsulation efficiency and Drug Loading Content

The encapsulation efficiency of PPADT-coated proanthocyanidins was calculated using UV-Vis absorption spectroscopy. 1 mL of the freshly prepared, undialyzed PRA@PPADT suspension was centrifuged at 2000 g for 5 min, and the supernatant was removed. A small amount of dichloromethane was added to the precipitate to ensure complete dissolution, followed by the addition of 1 mL of pure water. The mixture was placed in a fume hood and stirred overnight to allow complete evaporation of dichloromethane. Subsequently, the sample was centrifuged again at 2000 g for 5 min, and the supernatant was collected for UV-Vis measurement. The concentration of proanthocyanidins was calculated using a standard curve established with known concentrations. The encapsulation efficiency was calculated as the percentage of the encapsulated amount relative to the total initial input.

The drug loading of PRA@PPADT nanoparticles was determined via UV-Vis absorption spectroscopy. The freshly prepared PRA@PPADT suspension was dialyzed against ultrapure water for 24 h to remove unencapsulated free PRA, followed by lyophilization to obtain dry nanoparticle powder to constant weight. The accurately weighed nanoparticle powder was fully dissolved in a small amount of dichloromethane, and an equal volume of ultrapure water was added. The mixture was stirred overnight in a fume hood to completely evaporate dichloromethane. After centrifugation at 2000 g for 5 min, the supernatant was collected, and the encapsulated PRA concentration was quantified using a pre-established UV standard curve for PRA. The drug loading content was calculated as the mass percentage of encapsulated PRA relative to the total dry mass of nanoparticles, and three parallel replicates were tested for each sample.

4.8 ROS-Responsive Drug Release of PRA@PCT

Nile Red was encapsulated into nanoparticles as a model drug to simulate the drug release behavior of the nanoparticles. Nile Red-labeled nanoparticle suspensions (5 mg/mL) were incubated at 37 °C with PBS, simulated gastric fluid (SGF), simulated intestinal fluid (SIF) containing different concentrations of H_2_O_2_ (0 μM, 100μM, 1 mM, 10 mM) and ·OH (0.5 mM, 1 mM ), respectively. All samples were prepared in triplicate. At predetermined time intervals, 150 μL of the nanoparticle suspension was collected and transferred into a 96-well flat-bottom black polystyrene plate. The fluorescence intensity was measured at 607 nm with an excitation wavelength of 550 nm using a microplate reader. The cumulative Nile Red release was calculated according to the following equation: Cumulative Nile Red release (%) = (FL intensity(t_0_) − FL intensity(t)) / FL intensity(t_0_) × 100%, where t_0_ and t represent the initial time and each predetermined time interval, respectively.

PRA@PCT nanoparticles at a concentration of 1 mg/mL were first incubated in pH 1.2 buffer for 2 h to simulate gastric fluid conditions. Afterwards, the pH of the medium was adjusted to 6.8, followed by the addition of H_2_O_2_ to a final concentration of 100 μM. The mixture was incubated under constant shaking at 37 °C and 100 rpm. At each predetermined time point, aliquots were collected and centrifuged to harvest the supernatant, and the concentration of released PRA was quantified via UV-Vis spectrophotometry.

4.9 *In Vitro* Antioxidant Activity

The *in vitro* antioxidant activities of PRA@PCT nanoparticles at various concentrations were evaluated using three established methods: DPPH radical scavenging assay, ABTS^+^ radical cation scavenging assay (for total antioxidant capacity), and ·OH scavenging assay. All experiments were performed strictly following the instructions provided with the corresponding commercial assay kits, and each sample was tested in triplicate to ensure reliability. The results were expressed as mean values ± standard deviation (SD).

DPPH radical scavenging assay: The DPPH radical scavenging capacity of PRA@PCT was determined spectrophotometrically. A DPPH stock solution (0.1 mM) was prepared by dissolving 1.0 mg of DPPH in 19 mL of absolute ethanol under dark conditions. PRA@PCT was dissolved in absolute ethanol to prepare a series of concentrations (25, 50, 100, 200, and 400 µg/mL). For the assay, in a 96-well plate, 100 µL of each PRA@PCT solution was mixed with 100 µL of the DPPH stock solution. Control wells contained 100 µL of sample solution mixed with 100 µL of absolute ethanol (for sample background correction), and blank wells contained 100 µL of DPPH solution mixed with 100 µL of absolute ethanol. The plate was incubated in the dark at room temperature for 30 min, and the absorbance was measured at 517 nm. The DPPH radical scavenging rate was calculated according to the standard formula.

ABTS^+^ radical scavenging assay: The ABTS^+^ scavenging activity was measured using a Total Antioxidant Capacity (T-AOC) assay kit. PRA@PCT was dissolved in PBS to prepare a stock solution (5 mg/mL), which was subsequently diluted with PBS to generate a concentration series (25, 50, 100, 200, and 400 µg/mL). In a 96-well plate, 10 µL of each sample, 20 µL of the provided reagent four working solution, and 170 µL of the ABTS^+^ working solution were combined. For background correction, control wells contained 10 µL of sample mixed with 190 µL of PBS. The reaction proceeded in the dark for 6 min at room temperature, and the absorbance was measured at 405 nm. The ABTS^+^ scavenging rate was calculated according to the kit's instructions.

·OH scavenging assay: The ·OH scavenging capacity was assessed using a commercially available assay kit. PRA@PCT was dissolved in the specified assay buffer to prepare a series of concentrations (25, 50, 100, 200, and 400 µg/mL). Briefly, in reaction tubes, 0.2 mL of the substrate application solution was mixed with 0.2 mL of each PRA@PCT solution. Then, 0.4 mL of the provided reagent three application solution was added to all tubes, which were vortexed and incubated at 37°C for precisely 1 min. Immediately afterward, 2 mL of the chromogenic agent was added, and the mixture was vortexed again and kept at room temperature for 20 min. The absorbance was measured at 550 nm. Standard, blank, and sample control tubes were prepared according to the kit protocol for calibration and background subtraction. The ·OH scavenging rate was calculated using the formula provided with the kit.

4.10 Cytotoxicity Assay

Human normal colonic epithelial cells (NCM 460) and mouse monocyte-macrophage cells (RAW 264.7) were purchased from the American Type Culture Collection. NCM 460 cells were cultured in RPMI 1640 medium supplemented with 10% fetal bovine serum and 1% penicillin-streptomycin. RAW 264.7 cells were cultured in DMEM medium containing 10% fetal bovine serum. Both cell lines were maintained at 37 °C in a 5% CO_2_ atmosphere.

For cytotoxicity assays, NCM 460 and RAW 264.7 cells were seeded into 96-well plates at a density of 1×10^4^ cells per well, with five replicate wells per group. After cell attachment, the cells were incubated with various concentrations of PRA@PCT (12.5, 25, 50, 100, and 200 μg/mL) for 12 h. The old culture medium was then discarded, and the cells were rinsed three times with PBS. Subsequently, 100 μL of CCK-8 solution (diluted 1:10) was added to each well, followed by incubation in the dark for 1 h. The absorbance of each well at 450 nm was measured using a microplate reader, and cell viability for each group was calculated using the corresponding formula.

To establish a cellular oxidative stress model, RAW 264.7 cells were seeded into 96-well plates at a density of 1×10^4^ cells per well. After cell attachment, the culture medium was discarded, and the cells were gently washed once with PBS. Subsequently, H_2_O_2_ solutions diluted with PBS to various concentrations (200, 400, 800, and 1200 μmol/L) were added. Following incubation for 2 h, the medium was discarded, and the cells were washed twice with PBS. Cell viability was then determined using the CCK-8 assay.

4.11 Fluorescence Colocalization

For fluorescence colocalization, RAW 264.7 cells were first seeded in cell culture dishes at a density of 5×10^3^ cells/cm^2^. The cells were incubated with the prepared FITC/NileRed-PRA@PCT nanomedicine for 3 h, after which excess nanoparticles were removed. The RAW 264.7 cells were then treated with phosphate-buffered saline (10 μL), H_2_O_2_ (0.1 mM), or LPS (5 μg/mL) for 4 h, respectively.

4.12 *In Vitro* ROS Scavenging

For model establishment, lipopolysaccharide (LPS) and H_2_O_2_ were used to induce inflammatory and oxidative stress models, respectively, both of which are core pathological factors in the ulcerative colitis microenvironment. LPS activates the inflammatory response of RAW 264.7 macrophages through the TLR4/NF‑κB pathway, while H_2_O_2_ is used to induce oxidative damage in RAW 264.7 and NCM 460 cells.

RAW 264.7 cells were stimulated with various concentrations of H_2_O_2_ for 4 h, and cell viability was assessed using the CCK-8 assay to determine the optimal H_2_O_2_ concentration for subsequent experiments. A concentration of 200 μM H_2_O_2_ with a 4 h incubation was selected as the optimal modeling condition. To assess cell viability under oxidative stress, RAW 264.7 cells were stimulated with 200 μM H_2_O_2_ for 2 h, gently washed twice with PBS, and then treated with different concentrations of PRA@PCT (100 μg/mL and 200 μg/mL) for 4 h, followed by the CCK-8 assay.

In the *in vitro* cellular experiments, the free PRA group, PRA@PCT group and SP@PRA@PCT group were formulated to maintain an identical final equivalent concentration of PRA, with a unified final PRA concentration of 100 μg/mL. The administration concentrations of nanoparticles were converted into the corresponding PRA equivalents according to their drug loading capacities, namely PRA@PCT (2.5 mg/mL, normalized to PRA content) and SP@PRA@PCT (33 mg/mL, normalized to PRA content).

For intracellular ROS detection and live/dead staining, RAW 264.7 cells were seeded in 96-well plates at a density of 1×10^4^ cells per well. After cell attachment, the cells were treated with 200 μM H_2_O_2_ for 2 h, followed by incubation with PRA, PRA@PCT, or SP@PRA@PCT for 6 h. After incubation, the materials were removed, and the cells were gently washed three times with PBS. Intracellular ROS levels were assessed using the 2’,7’-dichlorodihydrofluorescein diacetate (DCFH-DA) probe. Cell viability was evaluated by Calcein-AM/PI staining and observed under an inverted fluorescence microscope.

For LPS-induced oxidative stress, RAW 264.7 cells were seeded in 96-well plates at a density of 1×10^4^ cells per well. After attachment, the cells were treated with 1 μg/mL LPS for 24 h. The old medium was then removed, and the cells were washed three times with PBS. Subsequently, the cells were incubated with SP, PRA, PRA@PCT, or SP@PRA@PCT for 6 h. After incubation, the materials were removed, and the cells were gently washed three times with PBS. Intracellular ROS levels were assessed using the DCFH-DA probe and observed under an inverted fluorescence microscope. Additionally, the changes in ROS levels were detected by flow cytometry.

4.13 Measurement of Mitochondrial Membrane Potential and Cellular ATP Content

NCM 460 cells were seeded into 96-well plates at a density of 1×10^4^ cells per well, and RAW 264.7 cells were seeded into 96-well plates at a density of 3×10^5^ cells per well. The cells were cultured overnight to allow attachment. RAW 264.7 cells were treated with 1 μg/mL LPS for 24 h. After incubation, the old medium was removed, and the cells were washed three times with PBS. Subsequently, the cells were co-incubated with PRA, PRA@PCT, or SP@PRA@PCT for 6 h. Following incubation, the materials were removed, and the cells were gently washed three times with PBS. The cells were then stained with JC-1 fluorescent probe for 20 min and observed under an inverted fluorescence microscope.

NCM 460 and RAW 264.7 cells were treated with 200 μM H_2_O_2_ for 2 h. After treatment, the old medium was removed, and the cells were washed three times with PBS. The cells were then incubated with PRA, PRA@PCT, or SP@PRA@PCT for 6 h. Following incubation, the materials were removed, and the cells were gently washed three times with PBS. The cells were stained with JC-1 fluorescent probe for 20 min and observed under an inverted fluorescence microscope.

Measurement of intracellular ATP levels was performed using an ATP Assay Kit (Beyotime). Briefly, RAW 264.7 cells were seeded in six-well plates and cultured overnight. The cells were treated with 1 mL of LPS (1 μg /mL) for 24 h. After treatment, the old medium was removed, and the cells were washed three times with PBS. Subsequently, the cells were incubated with PRA, PRA@PCT, or SP@PRA@PCT for 6 h. Following incubation, the materials were removed, and the cells were gently scraped off in a consistent direction along the well wall using a cell scraper. Then, 200 μL of lysis buffer was added to each well, and the collected samples were placed on ice. After lysis, the samples were centrifuged at 12000 g for 5 min at 4 ^o^C, and the supernatant was collected for subsequent analysis. An appropriate amount of ATP detection reagent was diluted with ATP detection reagent diluent at a 1:9 ratio. For ATP concentration measurement, 100 μL of ATP detection working solution was added to each assay well. The plate was incubated at room temperature for 3-5 min to deplete background ATP and reduce background signal. Subsequently, 20 μL of each sample was added to the wells, mixed quickly by pipetting, and after an interval of at least 2 s, the relative light unit (RLU) value was measured using a luminometer.

4.14 Apoptosis Assay

RAW 264.7 cells were seeded in six-well plates and cultured overnight. The cells were treated with 1 mL of LPS (1 μg/mL) for 24 h. After treatment, the old medium was removed, and the cells were washed three times with PBS. Subsequently, the cells were incubated with SP, PRA, PRA@PCT, or SP@PRA@PCT for 6 h. Following incubation, the cells were gently scraped off in a consistent direction along the well wall using a cell scraper. The cells were collected by centrifugation at 500 g for 5 min at 4 °C and washed twice with pre-cooled PBS, with centrifugation at 500 g for 5 min at 4 °C after each wash. The cells were then gently resuspended in pre-cooled 1× Binding Buffer. An aliquot of 100 μL of the cell suspension was taken, and 5 μL of Annexin V-FITC and 5 μL of propidium iodide (PI) were added. The mixture was gently vortexed and incubated at room temperature in the dark for 8-10 min. Subsequently, 400 μL of pre-cooled 1×Binding Buffer was added, and the samples were gently mixed. Apoptosis was detected by flow cytometry.

4.15 Cell Scratch Wound Healing Assay

NCM 460 cells were seeded in six-well plates at a density of 1×10^6^ cells per well. When the cell density reached approximately 90%, the cells were treated with 200 μM H_2_O_2_ for 2 h. After treatment, the old medium was removed, and the cells were washed three times with PBS. Subsequently, a wound was created by scratching perpendicular to the orientation line using a 200 μL pipette tip. The cells were washed three times with PBS to remove detached cells, and the initial wound width was observed and recorded under an inverted microscope. The cells were then incubated with PRA, PRA@PCT, or SP@PRA@PCT. At 24 and 48 h, images were captured under an inverted microscope. The wound distances for each group were quantified using Image J software, and the cell migration rate was calculated.

4.16 Analysis of Cellular Antioxidant and Anti-inflammatory Effects

RAW 264.7 cells were seeded in 6-well plates at a density of 1 ×10^6^ cells per well and cultured overnight. The cells were then stimulated with LPS (1 μg/mL) for 24 h. Following LPS stimulation, the cells were washed and treated for 6 h with free PRA, PRA@PCT, or SP@PRA@PCT. After the treatment period, the cell culture supernatants were collected and centrifuged to remove any debris. For the analysis of intracellular antioxidant status, the cells were washed, lysed, and the resulting lysates were used to determine the levels/activities of superoxide dismutase (SOD), malondialdehyde (MDA), catalase (CAT), and glutathione (GSH) using commercially available assay kits according to the manufacturers' instructions. Simultaneously, the levels of pro-inflammatory (IL-6, TNF-α, IL-1β) and anti-inflammatory (IL-10) cytokines in the collected cell culture supernatants were quantified using specific Elisa kits.

4.17 In *Vivo* Fluorescence Imaging

C57BL/6 male mice (6-8 weeks) were purchased from the Animal Experiment Center of Shanxi Medical University, and all procedures were conducted in compliance with the regulations of the Ethics Committee of Shanxi Medical University. Upon arrival, the mice were acclimatized for 7 days. They were randomly divided into three groups (n = 5 per group). The model group was provided with drinking water containing 2.5% DSS ad libitum for 7 days. Both normal and DSS-induced model mice were orally administered the ICG-labeled formulations. Mice in the normal group were intragastrically administered 200 μL of SP@PRA@PCT (3.3 g/kg). The PRA@PCT+DSS group received 200 μL of PRA@PCT (250 mg/kg), and the SP@PRA@PCT+DSS group received the same volume of SP@PRA@PCT (3.3 g/kg). *In vivo* NIR-II fluorescence imaging was performed at different time points using a small animal imaging system. Additionally, at 4, 8, 12, 24, and 48 h post-administration, the intestines and major organs of the mice were collected for fluorescence imaging observation.

4.18 Evaluation of Therapeutic Efficacy in DSS-Induced Ulcerative Colitis

Upon arrival, the mice were acclimatized for 7 days. They were randomly divided into 8 groups. Colitis was induced by providing mice with drinking water containing 2.5% DSS. To evaluate the therapeutic effect of SP@PRA@PCT, mice were orally administered water solutions of SP@PRA@PCT (PRA 10 mg/kg, volume 200 μL, once every 2 d) for 1 week. The mice in other groups were given equivalent SP, PRA, SP+PRA and PRA@PCT solutions as treated comparisons. In addition, we established a positive control group treated with 5-ASA (100 mg/kg), the standard first-line therapy for UC, for therapeutic efficacy comparison. Body weight, stool consistency, and fecal occult blood were recorded daily, and the disease activity index (DAI) score was calculated. On the final day of the experiment, fecal contents were collected under sterile conditions and immediately frozen in liquid nitrogen. Blood and major organs were collected, and the colon length was measured. Subsequently, colon tissue samples were fixed in 4% formalin (4% w/v paraformaldehyde) for H&E staining and histological analysis.

Paraffin-embedded colon tissues were used for immunofluorescence analysis of ZO-1 and Occludin. Specifically, embedded and fixed paraffin sections were deparaffinized and placed in distilled water. The sections were stained with hematoxylin solution to visualize nuclei and with alcoholic eosin solution to stain the cytoplasm. After dehydration, the sections were sealed with neutral resin. For immunofluorescence staining, paraffin sections were deparaffinized with xylene, rehydrated, and subjected to heat-mediated antigen retrieval using sodium citrate buffer (0.01 mol/L, pH 6.0). Subsequently, the sections were blocked with PBS (0.1 mol/L) containing 10% serum. The sections were incubated with primary antibodies against ZO-1 or Occludin overnight at 4 °C, followed by incubation with corresponding secondary antibodies. Finally, the nuclei were counterstained with DAPI (4’,6-diamidino-2-phenylindole), and the expression levels of the tight junction proteins ZO-1 and Occludin were observed using a microscope.

ROS levels in different colon tissues were detected using the dihydroethidium (DHE) method. Briefly, fresh colon tissues were immediately sectioned using a cryostat (thickness approximately 8-10 μm) without fixation to preserve ROS activity. The sections were mounted onto slides and rewarmed at room temperature for 10 min. DHE working solution (100 μmol/L) was applied, and the sections were incubated at 37 °C in the dark for 30-60 min. The sections were gently rinsed three times with PBS (pH 7.4) for 5 min each, avoiding strong light exposure. DAPI staining solution (2 μg/mL) was then applied, and the sections were incubated at room temperature in the dark for 5 min. After another three rinses with PBS, the sections were mounted with an antifade mounting medium and observed under a microscope.

Colon tissues were homogenized, and the protein concentration was determined using a BCA protein assay kit. The enzymatic activities and lipid peroxidation levels in the colon tissue homogenates, including SOD, MDA, CAT, and GSH were determined using corresponding assay kits. ATP levels in the tissues were measured using an ATP assay kit. The supernatant was collected, and the levels of inflammatory cytokines (IL-6, TNF-α, IL-1β, and IL-10) in the tissues were measured using Elisa kits.

4.19. Biosafety Evaluation

Collected blood samples were subjected to complete blood count and serum biochemical analyses. All harvested heart, liver, spleen, lung, and kidney specimens were processed for H&E staining.

4.20 Gut Microbiota Analysis

Collected fecal samples were used for gut microbiota analysis. Total genomic DNA was extracted from cecal contents using a commercial DNA extraction kit to ensure optimal yield and quality. The V3-V4 hypervariable region of the bacterial 16S rRNA gene, commonly used for microbial identification, was amplified via polymerase chain reaction (PCR) with region-specific primers. The resulting amplicons were subjected to high-throughput sequencing on an Illumina MiSeq platform. Sequencing libraries were established following standard protocols, and all bioinformatic analyses were conducted on the Majorbio Cloud Platform to assess the richness, diversity and shifts in intestinal microbial community structure across different mouse groups. These analyses included quality filtering, sequence alignment, and taxonomic classification, enabling the identification and quantification of bacterial taxa present in the cecal samples. Alpha diversity indices (e.g., Shannon, Simpson) were calculated to assess within-sample diversity, while beta diversity analysis (e.g., PCoA and NMDS) was performed to compare microbial community structures across different treatment groups. Community composition analysis (Venn diagrams, bar plots, heatmaps) was conducted to enumerate species (e.g., ASVs) shared among or unique to multiple groups or samples, facilitating the identification of distinctive species in disease groups. The relative abundance of microbial taxa in the model versus healthy groups was visualized to reveal trends in species distribution across different conditions. Species difference analysis (LEfSe) was performed to identify differentially abundant taxa across multiple taxonomic levels, with Linear Discriminant Analysis (LDA) scores used to estimate the effect size of each discriminant species.

4.21 Statistical Analysis

All data are presented as mean ± standard deviation (SD). Statistical significance was determined by one-way analysis of variance (ANOVA) using SPSS software, with significance levels denoted as ^*^*p* <0.05, ^**^*p* < 0.01, ^***^*p* <0.001, and n.s. indicating no significant difference.

**Figure S1.** (A) HPLC chromatogram of PRA standard; (B) HPLC chromatogram of the PRA sample.

**Table S1.** Linear relationship, recovery, and reproducibility of Cyanidin, Pelargonidin, and Peonidin

| Anthocyanin type | Linear regression  equation | R^2^ | Recovery(%) | RSD(%) |
| --- | --- | --- | --- | --- |
| Cyanidin | Y=118.52x+5.56 | 0.9996 | 98.7-100.31 | 0.54 |
| Pelargonidin | Y=72.49x-15.31 | 0.9998 | 96.4- 99.6 | 0.23 |
| Peonidin | Y=66.45x-13.14 | 0.9996 | 93.2- 97.5 | 0.82 |

**Table S2.** HPLC analysis of PRA

| Number | Anthocyanin type | Relative content (μg ⋅g^−1^) | **Proportion (%)** |
| --- | --- | --- | --- |
| a | Cyanidin | 53640 | 89 |
| b | Pelargonidin | 2950 | 5 |
| c | Peonidin | 3740 | 6 |

**Figure S2.** TEM micrograph of PRA.

**Figure S3.** ^1^H NMR spectrum of PPADT.

**Figure S4.** The encapsulation efficiency (EE) of PRA obtained with various oil phase-to-external water phase volume ratios

**Figure S5.** TEM images of PRA@PPADT fabricated at different oil phase and outer water phase

**Figure S6.** Hydrodynamic size distribution of PRA@PPADT.

**Figure S7.** 1H-NMR spectra of (A) TPP, (B) chitosan and (C) CS-TPP dissolved in D_2_O.

**Figure S8.** (A) UV-Vis absorption spectra and (B) FTIR spectra of CS, TPP, and CS-TPP.

**Figure S9.** (A, B) SEM image of SP. (C) Size distribution of microalgae. (D) Bright-field and (E) fluorescence micrographs of SP.

**Figure S10.** TEM images of SP@PRA@PCT.

**Figure S11.** (A) Fluorescence intensity of the Nile Red-loaded nanoparticle suspension; (B) Digital photographs showing the changes in transmittance of the nanoparticle solution after incubation with different concentrations of ·OH for 7 h. (C) Cumulative release profile of PRA from PRA@PCT triggered by 100 μM H_2_O_2_ in simulated intestinal fluid. Data are mean ± SD (n = 3). ^*^*p* <0.05, ^**^ *p* < 0.01, ^***^ *p* <0.001.

**Figure S12.** UV-Vis absorption spectra of the DPPH radical scavenging assay.

**Figure S13.** (A) Semi-quantitative analysis of intracellular ROS fluorescence intensity in RAW 264.7 cells macrophages. (B) Fluorescence microscopy images of ROS in H_2_O_2_-induced NCM 460 cells. Fluorescence microscopy images of ROS in H_2_O_2_-induced (C) RAW 264.7 cells and (D) NCM 460 cells. (E) Fluorescence micrographs of the mitochondrial membrane potential in NCM 460 cells. (F) Quantification of the ratio of mitochondrial aggregates to monomers. Data are mean ± SD (n = 3). ^*^*p* <0.05, ^**^ *p* < 0.01, ^***^ *p* <0.001.

**Figure S14.** (A) Dynamic changes in body weight of mice in each group over the experimental period. (B) DAI scores of mice in each group during the intervention period. (C) Quantitative analysis of colon length in each treatment group. (D) Representative images of stool consistency and rectal bleeding in each group. (E) Representative photographs of colons isolated from each group. (F) Representative H&E-stained sections of colon tissues from each group. Data are mean ± SD (n = 6). ^*^*p* <0.05, ^**^ *p* < 0.01, and ^***^ *p* <0.001.

**Figure S15.** Colonic histological damage scores. (A)Inflammation. (B) Ulceration depth. (C) Ulceration area. Data are mean ± SD (n = 3). ^*^*p* <0.05, ^**^ *p* < 0.01, ^***^ *p* <0.001, and n.s. = not significant.

**Figure S16.** Correlation heatmap between gut microbiota at genus level and colon-related indexes. Data are mean ± SD (Normal group: n = 6, DSS group: n = 5, SP group: n = 5, PRA group: n = 5, PRA@PCT group: n = 5 SP@PRA@PCT group: n = 4). ^*^*p* <0.05, ^**^ *p* < 0.01, ^***^ *p* <0.001, and n.s. = not significant.
